# Ce-Loaded HZSM-5 Composite for Catalytic Deoxygenation of Algal Hydrolyzed Oil into Hydrocarbons and Oxygenated Compounds

**DOI:** 10.3390/molecules27217251

**Published:** 2022-10-25

**Authors:** Mustafa Jawad Nuhma, Hajar Alias, Muhammad Tahir, Ali A. Jazie

**Affiliations:** 1 Department of Chemical Engineering, School of Chemical and Energy Engineering, Universiti Teknologi Malaysia, Johor Bahru 81310, Malaysia; 2 Chemical Engineering Department, College of Engineering, University of Al-Qadisiyah, Al-Diwaniyah City P.O. Box 88, Iraq; 3 Chemical and Petroleum Engineering Department, United Arab Emirates University (UAEU), Al Ain P.O. Box 15551, United Arab Emirates

**Keywords:** HZSM-5 zeolite, Cerium, Chlorella Vulgaris microalgae, deoxygenation, hydrocarbons, oxygenates

## Abstract

Despite the extensive research into the catalytic uses of zeolite-based catalysts, these catalysts have a limited useful lifetime because of the deactivating effect of coke production. This study looks at the use of Cerium (Ce) loaded HZSM-5 zeolite catalysts in the hydrocarbon and oxygenated chemical conversion from Chlorella Vulgaris microalgae crude oil. Characterization of structure, morphology, and crystallinity was performed after the catalysts were manufactured using the impregnation technique. Soxhlet extraction was carried out to extract the crude oil of microalgae. Transesterification reaction was used to produce algal hydrolyzed oil (HO), and the resulting HO was put to use in a batch reactor at 300 °C, 1000 rpm, 7 bars of nitrogen pressure, a catalyst to the algal HO ratio of 15% (wt. %), and a retention time of 6 h. To determine which Ce-loaded HZSM-5 catalysts would be most effective in converting algal HO into non-oxygenated molecules (hydrocarbons), we conducted a series of tests. Liquid product characteristics were analyzed for elemental composition, higher heating value (HHV), atomic ratios of O/C and H/C, and degree of deoxygenation (DOD%). Results were categorized into three groups: product yield, chemical composition, and carbon number distribution. When Cerium was added to HZSM-5 zeolite at varying loading percentages, the zeolite’s acid sites became more effective in facilitating the algal HO conversion. The results showed that 10%Ce/HZSM-5 had the greatest conversion of the algal HO, the yield of hydrocarbons, HHV, and DOD% (98.2%, 30%, 34.05 MJ/Kg, and 51.44%, respectively) among all the synthesized catalysts in this research. In conclusion, the physical changes seen in the textural characteristics may be attributed to Cerium-loading on the parent HZSM-5; nevertheless, there is no direct association between the physical features and the hydrocarbons yield (%). The primary impact of Cerium alteration of the parent HZSM-5 zeolite was to change the acidic sites required to boost the conversion (%) of the algal HO in the catalytic deoxygenation process, which in turn increased the hydrocarbons yield (%), which in turn increased the HHV and DOD%.

## 1. Introduction

Exploration into biofuels that can be made from both edible and non-edible biomass and that can be grown in several different ways has recently emerged as a promising avenue for addressing the world’s pressing energy needs [1,2,3,4,5,6]. The biggest problem, though, is that it uses up valuable farmland at a time when the world needs to focus on feeding its population. Microalgae have recently been suggested as one of the most promising prospects due to their photosynthesis, increased growth rate, ease of development, and, most importantly, the fact that their production does not take up valuable land that might be used to raise food. Algae also provide around half of the world’s oxygen while absorbing vast quantities of carbon dioxide. Microalgae are a potential and crucial renewable energy source because of their adaptability and high triglyceride content (up to 60%). Chlorella Vulgaris microalgae has great potential because of its high-fat content [7]. 

Drop-in biofuels (bio-hydrocarbons) are a possibility since they may be used in conventional vehicles in any mixture without needing modifications to engines or other mechanical parts and without disrupting the present fuel storage and distribution system. When compared to transesterification, the technology required to make biodiesel is more sophisticated and expensive [8]. As an alternate method of dealing with these problems, hydrodeoxygenation (HDO) has been developed. Numerous investigations have examined HDO at various hydrogen pressures and temperatures (from 10 to 40 bar and 260 to 350 °C, respectively), as well as with a wide variety of other operational factors, such as reaction duration, solvent usage, and catalyst to feed ratio [8,9,10,11,12,13]. HDO is a sort of hydrogenolysis that uses water to flush out oxygen molecules in lipids. HDO makes use of heterogeneous catalysts. The hydrodeoxygenation process (HDO) enables the synthesis of pure hydrocarbons that are fully compatible with conventional fuels, although the process is energy-intensive [14]. It takes at least 3–4 mol of hydrogen and a high hydrogen pressure of over 40 bar to completely deoxygenate 1 mol of reactants [15].

It has been suggested that the HDO process might be replaced by catalytic deoxygenation. However, hydrodeoxygenation, which produces H2O, can convert most of the carbon resources in the feedstock into hydrocarbons, while decarboxylation and decarbonylation both produce CO2 and CO, respectively, and thus result in a partial loss of the carbon resources contained in the triglyceride feedstock [14]. In addition, the catalyst is not destroyed as it is in hydrodeoxygenation, so no water is produced [16].

Catalytic cracking is one method developed to transform biodiesel from plant oils. Catalytic cracking breaks down long hydrocarbon chains into lighter fractions, while decarboxylation and decarbonylation remove oxygen molecules in the form of CO_2_, CO, or H_2_O [17]. The catalyst lowers the reaction temperature and boosts output [18]. Particle size, porous structure, acidity, and surface area are all factors that affect reaction pathway selectivity and product yield [19].

It was reported that zeolite catalysts were the most widely used catalysts in the upgrading of vegetable oil and bio-oil. Though HZSM-5 was touted as having the greatest performance for catalytic cracking of bio-feedstocks, many zeolite-based catalysts had a limited lifetime because of coke formation deactivation [20].

HZSM-5’s well-designed architecture facilitates the diffusion of most oxygenates to the pore-passage active acid sites [21]. Furthermore, the HZSM-5 catalyst has both Bronsted and Lewis acid sites, which are important in acid-catalyzed reactions. The -OH groups at Bronsted acid sites are produced during thermal treatment. Furthermore, Bronsted acid sites are more likely to be engaged in catalytic deoxygenation reactions due to the nature of the various intermediary steps involved [21,22,23].

Active components in the catalytic upgrading of oxygenates include transition metals including nickel (Ni), molybdenum (Mo), zinc (Zn), and iron (Fe) [23,24]. Transition metal-modified HZSM-5 catalysts are prone to coke production, despite their outstanding effectiveness in upgrading oxygenates to hydrocarbons [23].

Until then, we are unaware of any research examining the deoxygenation of bio-oils or FAMEs using a Cerium-modified HZSM-5 zeolite in a catalytic process. Surprisingly, studies show an interest in impregnating HZSM-5 with rare earth metals to catalytically convert oxygenated pyrolysis gases into hydrocarbons with reduced coke production [25,26,27,28]. For instance, Sun et al. studied the ethylation of benzene with ethanol in a fixed-bed reactor using zeolites modified with rare earth metals (La and Ce). Researchers found that there were somewhat fewer strong Bronsted acid sites, which inhibited coke formation [29].

Particularly strong acid sites trigger coke deposition, which in turn renders a catalyst ineffective. Additionally, Isha and Williams argue that cerium’s oxygen-storing properties mitigate the production of coke [25]. Researchers found that the smaller size of rare earth metal particles allowed them to access more of the zeolite channel and reduced the number of highly acidic Bronsted sites [26,27]. The thermal stability of the catalyst is enhanced by impregnating HZSM-5 with rare earth metals [27,28]. Hydrogen atom transfer, manipulated hydrocarbon synthesis, and reduced oxygenated compound output are all the result of rare earth metal ion exchange inside the zeolite framework.

Recent research indicates that rare earth metal has the potential to reduce coke concentration and improve fuel quality by catalytic deoxygenation of oxygenated molecules. More oxygenated molecules can be converted into hydrocarbons thanks to the rare earth metal’s ability to reduce coking on the catalyst’s surface and lengthen the catalyst’s useful life. Consequently, Cerium is further explored in this work as a bi-functional catalyst for catalytic deoxygenation upgrading of algal HO that is individually loaded with varied loading percentages on the parent HZSM-5. Accordingly, Cerium was loaded into the parent HZSM-5 framework for this research to improve the conversion of oxygenates into hydrocarbons during catalytic deoxygenation and to reduce the occurrence of coke. There is no comprehensive literature review that we could find on the effect of Ce/HZSM-5 on the enhancement of algal HO catalytic deoxygenation. In addition, the vast majority of the published work on modifying HZSM-5 with rare earth metals has concentrated on model compound catalytic cracking processes for various reactants other than algal HO (FAMEs), including alcohol [30,31,32], methyl mercaptan [33,34], alkane [35], pyrolysis of bio-mass [36,37], and furans [38].

The feasibility of using a Ce-loaded HZSM-5 zeolite catalyst for upgrading crude oil to more valuable hydrocarbons is being explored. X-ray diffraction (XRD), Nitrogen adsorption isotherms, Scanning Electron Microscopy (SEM), Temperature Programmed Desorption of Ammonia (NH_3_-TPD), and Thermogravimetric Analysis (TGA) were used to analyze four catalysts (parent HZSM-5, 5%Ce/HZSM-5, 10%Ce/HZSM-5, and 15%Ce/HZSM-5). In a batch reactor, we tested how well several synthetic catalysts deoxygenated oil extracted from the microalgae Chlorella Vulgaris. To improve the catalytic deoxygenation of the algal HO and therefore convert the oxygenated molecules into hydrocarbons, the effect of Cerium loading weight percentages on the parent HZSM-5 was studied. The chemical components of products were analyzed and identified using gas chromatography-mass spectrometry (GC-MS). Product yield, chemical composition, and carbon number distribution were the three categories into which the results were sorted. Analysis of the liquid product included determining its constituent make-up, higher heating value (HHV), O/C and H/C atomic ratios, and degree of deoxygenation (DOD%).

## 2. Experimental

### 2.1. Materials

The following materials were used for the synthesis of HZSM-5, Ce/HZSM-5 catalysts, and used also to extract and hydrolyze the crude oil of Chlorella Vulgaris microalgae as follows: ZSM-5 in Ammonium form [NH_4_^+^] (Si/Al = 30) with a purity of 100 %, from Alfa Aesar (Haverhill, MA, USA). Cerium nitrate hexahydrate (Ce(NO_3_)_3_.6H_2_O) from Alfa Aesar (USA) is also available in 99.9% purity. Chlorella Vulgaris microalgae powder was purchased from FocusHerb LLC, China. Methanol from Hayman Company with a purity of more than 99.85% (London, UK), Hexane from Thomas Baker with a purity of 95 % (Mumbai, India), Sulfuric acid from Chem-Lab NV with a concentration of 96% (Zedelgem, Belgium), and Sodium hydroxide pellets from Scharlau (Sentmenat, Spain).

### 2.2. Extraction of the Crude Oil from Chlorella Vulgaris Microalgae

Using a soxhlet extractor technique, crude oil from Chlorella Vulgaris microalgae was extracted. The initial purpose of the soxhlet extractor was to facilitate the removal of lipids from solids. Soxhlet extraction is the method of choice when the target molecule is poorly soluble in a solvent and the impurity is insoluble in that solvent. The soxhlet extractor system (shown schematically in Figure S1 in the Supplementary Materials) recycles hexane by continuously boiling and condensing it. The algae reservoir was filled with powdered Chlorella Vulgaris microalgae, and the hexane/oil reservoir was filled with 200 mL of liquid hexane. In this case, the hexane completely submerges the microalgae powder and dissolves some of it. Once the algae reservoir is full, the hexane forms a siphon and drains into the hexane/oil reservoir, taking any dissolved oil with it. The hexane and oil mixture was heated to 110 °C on a hot plate. Due to its higher boiling point, oil does not evaporate during the boiling process. Therefore, the hexane can climb via the tubes shown by the dashed line in Figure S1 (in the Supplementary Materials). Cooling water that surrounds the condenser tube takes heat from the vapor hexane, allowing it to condense when it reaches the tube’s interior. As the hexane condenses, it flows into the algae reservoir, where it dissolves additional oil and submerges the microalgae powder. At a temperature of 110 °C, the recirculation process will run for a full eight hours. At the completion of the extraction procedure, a hexane and oil mixture remained in the hexane/oil reservoir, and the hexane-soaked filter papers with the remaining microalgae powder were placed in another algae reservoir. To separate the hexane from the crude oil of Chlorella Vulgaris microalgae, the mixture was collected from the hexane/oil reservoir at the end of the extraction period (8 h), stored safely in the flask, and then delivered to the rotary evaporator. The hexane and oil combination is stored in the hexane and oil reservoir of the rotary evaporator device, heated to 74 °C to separate the hexane, and then the vaporized hexane is condensed and returned to the hexane reservoir through a condenser [39].

### 2.3. Hydrolysis of the Crude Oil of Chlorella Vulgaris Microalgae

Fatty acid methyl esters (FAMEs) were synthesized from algal hydrolyzed oil of Chlorella Vulgaris microalgae (HO) utilizing a transesterification method. A hydrolysis setup, including a distillation column and a 250 mL round-bottom flask with a stirrer, will be used to carry out the transesterification process. The flask will be heated in a water bath. First, you’ll need to measure out 5 g of the crude oil and heat it to 48 °C while stirring at 300 rpm for 10 min. Concurrently, 110 mL of methanol was added to 4.500 g of solid NaOH and agitated until the particles of sodium hydroxide were completely dissolved, yielding a methoxide (methanol–sodium hydroxide) solution. As the crude oil for Chlorella Vulgaris microalgae is low in lipid weight, the amount of this solution (methoxide) was increased to be around 20 times greater than the crude oil weight; 100 g of the methoxide solution was added to the heated crude oil, and the resulting mixture was heated to 48 °C for 1 h while being stirred at 400 rpm in a round bottom flask [40]. The mixture was neutralized to PH = 7 using diluted sulfuric acid H_2_SO_4_ (1 M), and then it was transported to the separating funnel for 10 h after the flask temperature had cooled to room temperature. Separating funnels create two distinct levels. FAMEs make up the top layer, whereas glycerol is found at the bottom. 

### 2.4. Catalyst Preparation

To prepare the parent (HZSM-5) zeolite catalyst as well as three Cerium-modified variants (5%Ce/HZSM-5, 10%Ce/HZSM-5, and 15%Ce/HZSM-5). The ammonium form of ZSM-5 (NH_4_^+^) was calcined at 600 °C for 4 h in static air (Ramp rate = 5 °C/min) to produce the proton form of zeolite, HZSM-5 [21]. An incipient wetness methodology, with minor modifications to the method published by Dueso et al. [41], was employed for the impregnation of Cerium rare earth metal on the support (HZSM-5) at various weight percents. For example, 10 g of 5%Ce/HZSM-5 catalyst can be made by combining 9.5 g of HZSM-5 with 250 mL of deionized water, followed by 1.54 g of Ce(NO_3_)_2_.6H_2_O (Cerium nitrate hexahydrate) and allowing the mixture to sit at room temperature for 1 h.

The resulting slurry was stirred continuously at a steady temperature of 90 °C until all of the water evaporated, forming a paste. After drying the paste at 110 °C for a whole night in an oven, it was calcined at 750 °C for three hours to eliminate any remaining impurities [42], before being cooled to room temperature in a desiccator. As a final step, the produced catalysts were crushed using a crusher and pestle before being carefully put away for subsequent use in this investigation. The additional catalysts were prepared in the same way; both 10%Ce/HZSM-5 and 15%Ce/HZSM-5 are available.

### 2.5. Catalyst Characterization

The characterizations were performed to investigate the physicochemical properties of synthesized catalysts. All methods of characterization were carried out by standard procedures. X-ray diffraction was used to determine the degree of crystallinity in the catalysts (XRD). Powder XRD patterns were obtained using a PW1730 diffractometer (Philips, USA) at 40 kV and 30 mA with Cu as the anode material (k = 1.54 Å). The scanning step size was 0.04°/min, with 1 s between steps, within a 2θ range of 5–100°.

At −196 °C, an adsorption isotherm for nitrogen was measured using a BELSORP-mini II (BEL Japan Inc., Osaka, Japan). The samples were degassed at 200 °C under a vacuum for 6 h to eliminate any adsorbed chemicals before analysis. Based on the Brunauer–Emmett-Teller (BET) and t-plot models, the specific surface area and porosity were determined. At p/p_0_ = 0.95, the total pore volumes were determined. Micropore and mesopore surface area and pore size distribution were calculated using the Barrett–Joyner–Halenda (BJH) method.

Scanning electron microscopy (MIRA III, TESCAN, Brno, Czech Republic) with an energy-dispersive X-ray spectroscopy (EDS) detector (SAMX, France) was used to characterize the surface morphology and crystal size of the zeolites.

The samples’ acidity was measured with a NanoSORD-NS91 (Sensiran Co., Shiraz, Iran) analyzer using the Temperature Programmed Desorption of Ammonia (NH_3_-TPD) method. 

All freshly synthesized catalysts were subjected to Thermogravimetric Analysis (TGA) with Q600 (TA, USA) in an air atmosphere at a heating rate of 20 °C/min between temperatures of 40 and 800 °C to determine the likelihood of coke formation on the catalysts. Q600 (TA, USA) instrument was used to conduct air-atmosphere thermal gravimetric analysis (TGA) of the parent zeolite HZSM-5 and Cerium (Ce) modified HZSM-5 zeolite catalysts with different loading weight percent of Ce (5, 10, and 15). The sample was applied to the instrument and kept at 40 °C for 1 min. The sample was then heated to 720 °C at a rate of 20 °C/min in dry air at a flow rate of 100 mL/min. The rate of change in mass as a function of temperature (dm/dT, %/°C) was determined experimentally.

### 2.6. Catalytic Deoxygenation of the Alga HO Using the Parent HZSM-5, and Lanthanum Modified Zeolites

**Figure 1:**
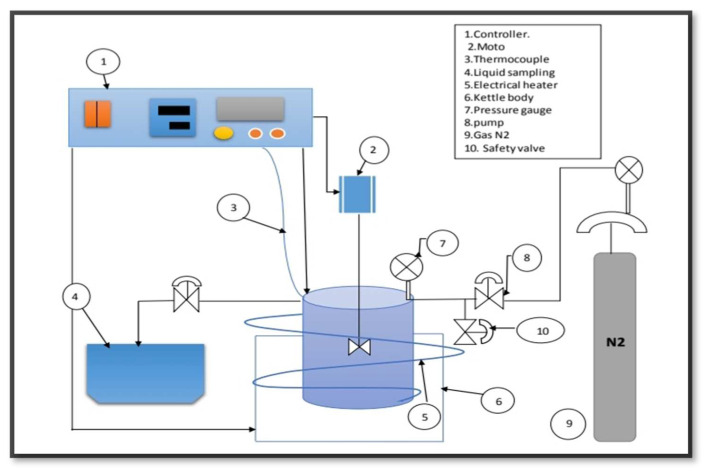
Schematic diagram of the used batch reactor.

**Figure 2:**
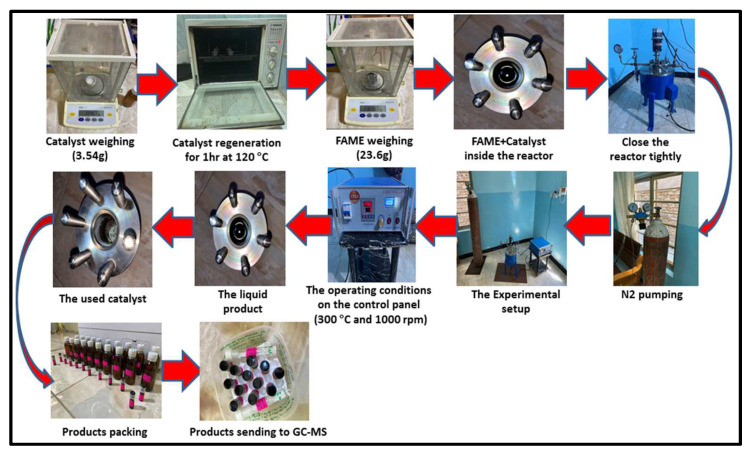
Experimental images for the procedure of conducting the catalytic deoxygenation reactions for the hydrolyzed oil of Chlorella Vulgaris microalgae.

As can be seen in Figure 1, the algal HO was catalytically deoxygenated in a 100 mL stirring batch reactor (Zhengzhou Keda Machinery and Instrument Co., Tianjin, China) (ZZKD). After 23.6 g of algal HO and 3.54 g of catalyst were combined, the resulting mixture was placed into the reactor. The air was forced out of the reactor by compressing 5 bar of Nitrogen (N_2_) three times; the initial N_2_ pressure was then compressed to 7 bar and held in the reactor. The impeller in the reactor was set to 1000 rpm, and the temperature was maintained at 300 °C for 6 h. When the reaction was completed, the mixtures were allowed to cool to room temperature. There was a discharge of the gaseous phase (not studied). The catalyst was isolated via filtration from the liquid phase. Figure 2 shows the results of an analysis performed on a sample of the liquid product.

### 2.7. Product Analysis

Gas chromatography-mass spectrometry (GC-MS) was used to determine the chemical components of the algal HO and the liquid product, with the gas chromatography system being an Agilent Technologies 7820A GC System outfitted with a mass selective detector GC-5977E MSD operating in electron ionization (EI) mode at 70 eV. The column employed was a 100% dimethylpolysiloxane Ultra Alloy Capillary Column UA-5MS (P/N UA1-30M-1.OF, Frontier Laboratories Ltd., Fukushima, Japan) with a 250 μm inner diameter, a 0.25 μm film thickness, and a 30 m working length. The oven temperature was kept at 45 °C for 1 min before being increased to 300 °C at a rate of 6 °C/min for 40 min. The GC-MS spectra were compared to the NIST mass spectral database to determine the components of the final product. 

The peak area percentage of the GC-MS chromatogram, which may also be expressed as yield percentage, can be used to compute the relative fraction of product chemicals [43], which was shown to be Equation (1). The conversion percentage for the algal HO (Table 1) was calculated by using Equation (2) [44], with the same method described by Katikaneni et al. [45]. The average content (wt. %) of X (X = C, H, and O) was calculated by Equation (3) [46]. The higher heating value (HHV) of the product was calculated by using Equation (4) [46,47]. Equation (5) is used to compute the degree of deoxygenation (DOD %) [48].

(1) $$\mathrm{Yield}\;\left(\%\right)=\frac{\mathrm{Area}\;\mathrm{of}\;\mathrm{the}\;\mathrm{desired}\;\mathrm{product}}{\mathrm{Area}\;\mathrm{of}\;\mathrm{all}\;\mathrm{detected}\;\mathrm{substances}}\times 100$$

(2) $$\mathrm{Conversion}\;\left(\%\right)=\frac{\mathrm{Mass}\;\mathrm{of}\;\mathrm{initial}\;\mathrm{compound}\;\mathrm{in}\;\mathrm{the}\;\mathrm{HO}-\mathrm{Mass}\;\mathrm{of}\;\mathrm{the}\;\mathrm{compound}\;\mathrm{in}\;\mathrm{the}\;\mathrm{product}}{\mathrm{Mass}\;\mathrm{of}\;\mathrm{initial}\;\mathrm{compound}\;\mathrm{in}\;\mathrm{the}\;\mathrm{HO}}\times 100$$

(3) $$\mathrm{wt}.\;\%\;X\;=\frac{\mathrm{Mass}\;\mathrm{of}\;X\;\mathrm{in}\;\mathrm{products}}{\mathrm{Mass}\;\mathrm{of}\;\mathrm{products}}$$

(4) $$\mathrm{HHV}\;\left(\frac{\mathrm{MJ}}{\mathrm{Kg}}\right)=-1.36\;75+0.3137\;\left(C\right)+0.7009\;\left(H\right)+0.0318\;\left(O\right)$$

(5) $$\mathrm{DOD}\%\;=\frac{\mathrm{Molar}\left(\frac{O}{C}\right)\mathrm{of}\;\mathrm{the}\;\mathrm{algal}\;\mathrm{HO}\;\mathrm{in}\;\mathrm{feed}\;-\;\mathrm{Molar}\;\left(\frac{O}{C}\right)\mathrm{of}\;\mathrm{the}\;\mathrm{catalytic}\;\mathrm{cracking}\;\mathrm{products}}{\mathrm{Molar}\left(\frac{O}{C}\right)\mathrm{of}\;\mathrm{the}\;\mathrm{algal}\;\mathrm{HO}\;\mathrm{in}\;\mathrm{feed}}$$

## 3. Results and Discussions

### 3.1. Catalyst Characterization

#### 3.1.1. XRD Results

**Figure 3:**
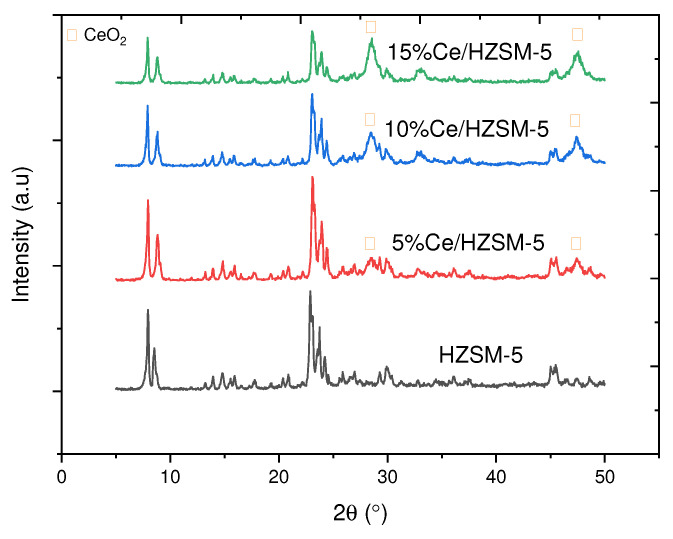
XRD patterns for parent HZSM-5 and Cerium-modified zeolites catalysts.

Both the phase purity and crystallinity of the original HZSM-5 and the 5%Ce/HZSM-5, 10%Ce/HZSM-5, and 15%Ce/HZSM-5 produced catalysts were evaluated using X-ray diffraction (XRD) analysis, as shown in Figure 3 and Table 1. Therefore, the parent HZSM-5 zeolite samples have a typical MFI-type structure (mordenite framework inverted) as shown by the XRD peaks at (2θ = 7.96, 8.52, 14.8, 22.88, 24.24, 29.92, and 45.48°) [31,33,36,49,50]. The peaks at the typical angles were found to be slightly off from the reference standards, and this discrepancy was ascribed to the various X-ray sources used [51].

The crystallographic structure of MFI was not significantly changed after loading Cerium and calcination, as evidenced by the fact that the main diffraction intensities of the synthesized Cerium-modified catalysts (2θ = 7–9° and 23–25°) correspond to the main diffraction intensities of the parent HZSM-5 catalyst [21,33]. Probably because such low levels of Cerium loadings are distributed strongly over the support with extremely tiny particles, very weak diffractions corresponding to CeO_2_ crystallite were found at loadings of 5 wt. %. CeO_2_’s distinctive diffraction peaks (2θ = 23.08°, and 45.52°) were seen at 10 and 15 wt. % Cerium loadings, and their diffraction strengths rose with increasing Cerium loadings [52]. The supported metal catalysts on HZSM-5 exhibited a size-dependent growth in the crystallite size of metal oxide with increasing metal loading concentration [53]. CeO_2_ crystallite sizes increased with increasing Cerium loadings, leading to stronger diffraction peaks [21,38,54].

Consistent with the findings of Balasundram et al. [21], it was shown that the 5%Ce/HZSM-5 catalyst exhibits stronger diffraction peak intensities than the original HZSM-5 catalyst. The increased absorption coefficient of the metals during calcination, which varies with loaded metal concentration, may account for the variations in XRD peak intensities for metal-laden HZSM-5 catalysts [22].

The XRD patterns for 10%Ce/HZSM-5 and 15%Ce/HZSM-5 demonstrate that the peak intensity gradually decreases with increasing Cerium content, and thus the crystallinity gradually decreases with increasing Cerium contents. This phenomenon was possibly due to the increase in the coverage of Cerium species on HZSM-5 and/or the reduction in the crystallinity with the increase in Cerium-loading [38]. When any species is present inside the channels, the XRD intensities in the HZSM-5 pattern will change accordingly [55]. This suggests that Cerium species are entering the HZSM-5 channels, as seen by a steady drop in peak intensity in the patterns of 10%Ce/HZSM-5 and 15%Ce/HZSM-5 [38,56].

As shown in Table 1, the relative crystallinity of the 5%Ce/HZSM-5, 10%Ce/HZSM-5, and 15%Ce/HZSM-5 was calculated using the peak area between 2θ = 22.5−25° [57,58] as a function of the crystallinity of the parent HZSM-5 catalyst as a standard. This table shows that as the percentage of Cerium inside the channels of the parent HZSM-5 increases, the relative crystallinity of the material decreases, with 5%Ce/HZSM-5 exhibiting higher relative crystallinity than the parent material and 10%Ce/HZSM-5 exhibiting a higher relative crystallinity than 15%Ce/HZSM-5.

#### 3.1.2. Surface Analysis

Table 2 displays the textural features of the original HZSM-5 and the Cerium-modified HZSM-5 zeolite catalysts with varying loading weight percents of Cerium (5, 10, and 15%). Table 2 shows that when the fraction of Cerium loaded onto the parent HZSM-5 catalyst increased, the values of all of these textural qualities reduced significantly. The BET surface area of 5%Ce/HZSM-5, 10%Ce/HZSM-5, and 15%Ce/HZSM-5 was 308 m^2^/g, 286 m^2^/g, and 258 m^2^/g, respectively, whereas it was 338 m^2^/g for the parent HZSM-5. In comparison to the parent HZSM-5, which had a micropore area of 195 m^2^/g, the micropore areas of 5%Ce/HZSM-5, 10%Ce/HZSM-5, and 15%Ce/HZSM-5 were 166, 154, and 142 m^2^/g, respectively. The total surface area of HZSM-5 was reduced from 143 to 142, 131, and 115 m^2^/g, reflecting a similar trend in the parent compound. When 5%Ce was added to HZSM-5, the total pore volume dropped to 0.2 cm^3^/g, 10%Ce to 0.19 cm^3^/g, and 15%Ce to 0.17 cm^3^/g. The loading weight % increases as the accumulated Cerium oxides do on the pore mouth of the Cerium-modified zeolite catalysts, which might explain why the original HZSM-5’s textural qualities are diminished relative to those of the Cerium-modified HZSM-5. To a greater extent, the values of all these textural characteristics decrease when cerium cations, which are smaller in size, diffuse through the pore mouth of HZSM-5 and subsequently deposit inside the internal pore channel of the parent HZSM-5 catalyst [21,29,59,60]. Furthermore, it can validate the hypothesis of the reduced XRD intensities shown by the XRD pattern [see Figure 3] [33]. As can be shown in Table 2, increasing the Cerium loading weight percentage results in a larger average particle size for the parent HZSM-5, which rose from 17 nm for 5%Ce/HZSM-5 to 19, 20, and 23 nm for 10%Ce/HZSM-5 and 15%Ce/HZSM-5, respectively. The average particle size of Cerium-doped catalysts has grown because the loaded Cerium has been deposited on the crystal surfaces, covering more of the crystal’s surface area [35].

**Figure 4:**
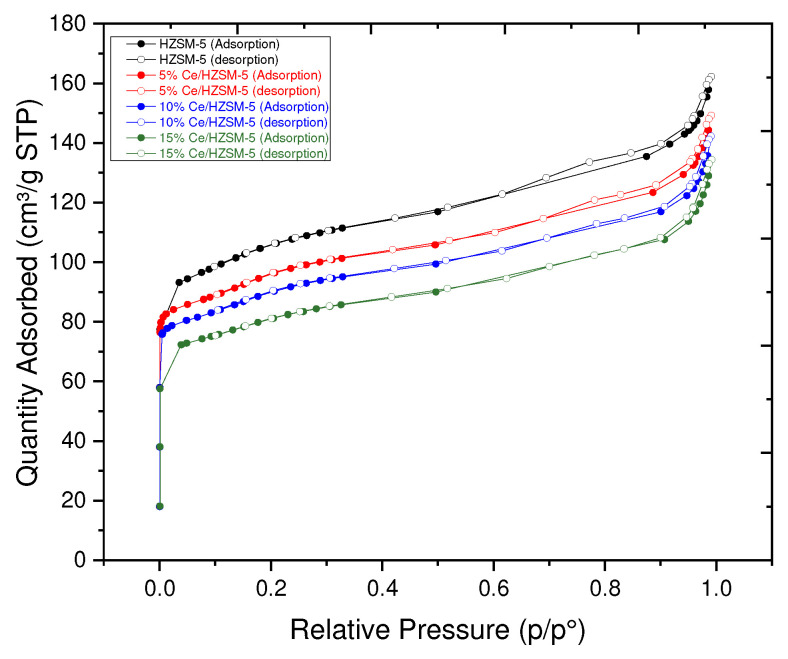
N_2_ adsorption–desorption isotherms of the parent HZSM-5 and Cerium-modified HZSM-5 with different loading weight percentages.

As is shown in Figure 4, Similar to HZSM-5, the N_2_ adsorption–desorption isotherms of Cerium-modified zeolite catalysts are found to be of type I isotherms with an H_3_ hysteresis loop at a high P/P_0_ region, characteristic of micropore and mesoporous materials [33,61]. In addition, the N_2_ adsorption was shown to be inversely proportional to relative pressure over the whole pressure range (P/P_0_) when the Cerium loading weight percent was increased on the parent HZSM-5 catalyst. The creation of a microporous zeolite material was verified by the significant rise in N_2_ adsorption over the samples in the low and medium pressure areas P/P_0_ (0–0.35) [32].

The existence of slit-shaped holes is indicated by a shift in the absorption of nitrogen (N_2_) at a relative pressure (P/P_0_) between 0.35–0.9 [62,63], with the hysteresis loops resulting from capillary condensation inside mesopores through nitrogen multilayers adsorbing on the inner surface [64].

In addition, HZSM-5 and Cerium-modified catalysts have the same property of interparticle macroporosity, namely a minor N_2_ uptake step at a relative pressure (P/P_0_) in the range of 0.900–1.00 [34]. Both the original HZSM-5 and the Ce/HZSM-5 modified catalysts exhibit micropores and mesopores. 

#### 3.1.3. Ammonia TPD Analysis

**Figure 5:**
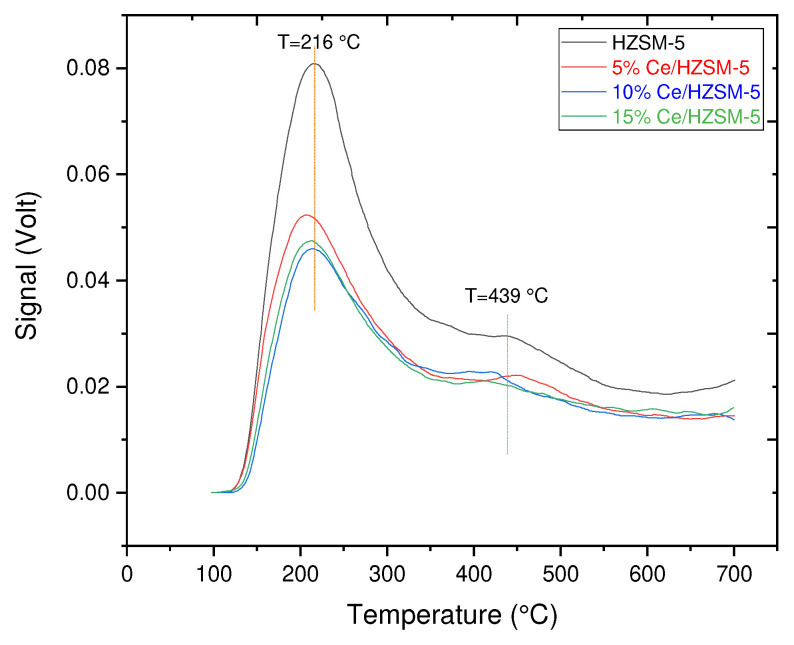
NH_3_-TPD profiles of the parent HZSM-5 and Cerium-modified catalysts: 5%Ce/HZSM-5, 10%Ce/HZSM-5, and 15%Ce/HZSM-5.

NH_3_-TPD was used to characterize weak acid sites and strong acid sites on parent HZSM-5 and Cerium modified HZSM-5 zeolites catalysts (5%Ce/HZSM-5, 10%Ce/HZSM-5, and 15%Ce/HZSM-5) [65,66]. In Figure 5, NH_3_-TPD profiles for both the parent HZSM-5 and the Cerium-modified HZSM-5 zeolites are shown. The parent HZSM-5 catalyst had a typical NH_3_-TPD profile, with two maximal peaks at low and high temperatures. The desorption of NH_3_ from Lewis sites (weak acid sites) (such as extra-framework aluminum) has been attributed to the low-temperature desorption peak at 216 °C [67,68], while the desorption of NH_3_ from the Bronsted acid sites (strong acid sites) deriving from framework aluminum has been attributed to the high-temperature desorption peak at 439 °C [69,70,71]. Figure 5 shows that the peak areas indicated the number of acid sites and that the peak temperatures were assigned the acid strength [34]. Figure 5 also demonstrates that the total acidity of the synthesized Cerium modified HZSM-5 zeolite catalysts is lower than that of the parent HZSM-5 as the Cerium loading (wt. %) on the parent HZSM-5 increases. This is in agreement with the findings of previous researchers who explained that the modification of zeolites with metals alters the total acidity of the zeolites [33,54,72].

The surface acidity of the catalysts may be modified by the incorporation of Cerium species into the parent HZSM-5 zeolite tunnel by dispersion or exchange with H^+^, converting some of the strong Bronsted-acid (B) centers into Lewis-acid (L) centers [73,74,75,76]. In addition, Lewis acid sites in zeolite catalysts are often linked with non-framework (extra-framework) aluminum species [77], whereas aluminum in the zeolitic framework may create strong acid sites [78]. Thus, the higher proportion of Lewis acid sites relative to Bronsted-acid sites in the doped catalysts indicated a higher concentration of non-framework aluminum species. In the meantime, the XRD data implies that dealumination in the HZSM-5 framework is responsible for the increased number of non-framework aluminum species. As a result, the concentration of strong acid sites may be lowered by the dealumination of the framework aluminum species. Reducing the concentration of strong acid sites helps prevent the production of coke deposits, as shown by Ouyang et al. It has also been shown that reducing the number of strong acid sites stabilizes the catalyst’s active sites [30].

Figure 5 shows that the low-temperature peak of all Cerium-modified zeolite catalysts is shifted to lower temperatures, with a similar peak profile compared with the peak temperatures of the parent HZSM-5 (at 216 °C for the parent HZSM-5), while the high-temperature desorption peak clearly decreases, and is shifted to lower temperatures peak (except for the case 5%Ce/HZSM-5).

Table 3 displays the results of using calibration curves of TCD values in Volt (V) to determine desorbed NH_3_ levels in mmol/g. The total acidities of the Cerium-modified zeolites catalysts were found to decrease from 0.740 mmol/g for the parent HZSM-5 to 0.490 mmol/g for 15%Ce/HZSM-5 as the Cerium loading weight percentage was increased, indicating that the loading of Cerium affects acidic characters of the parent HZSM-5 catalysts and thus led to the decrease of both strong and weak acid sites amounts.

#### 3.1.4. Thermogravimetric Analysis

**Figure 6:**
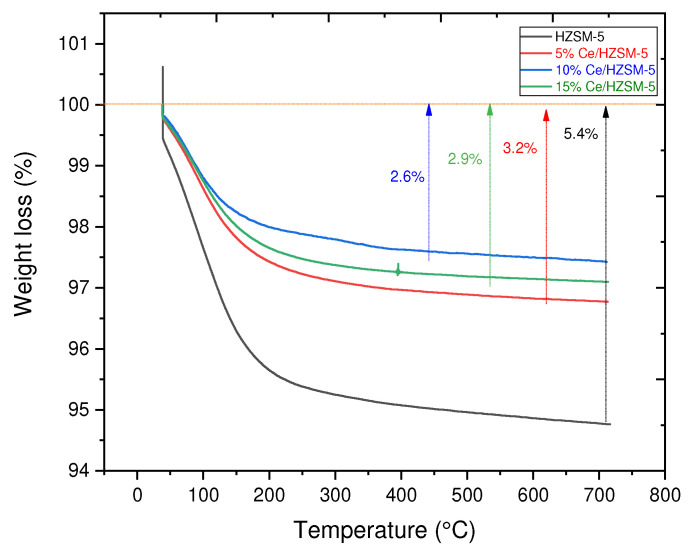
TGA of the fresh parent HZSM-5 and fresh Cerium modified HZSM-5 with different loading weight percentages.

In this study, Thermogravimetric analysis (TGA) was utilized to estimate the quantity of carbon that would fill the pores of freshly synthesized catalysts employed in the catalytic deoxygenation process in this study [79]. Thermogravimetric analysis (TGA) results for all freshly synthesized catalysts are shown in Figure 6. The total mass loss for HZSM-5 is 5.4%, while it is 3.2%, 2.6%, and 2.9% for 5%Ce/HZSM-5, 10%Ce/HZSM-5, and 15%Ce/HZSM-5, respectively, apparently due to the release of water from narrow channels [80,81], especially in the range of (30–170 °C), which accompanied water removal from the fresh samples [82]. In conclusion, compared to the original HZSM-5, the catalyst’s capacity to generate a coke deposit is reduced when Cerium is added. 10%Ce/HZSM-5 had the least amount of mass annihilation.

#### 3.1.5. SEM Analysis

**Figure 7:**
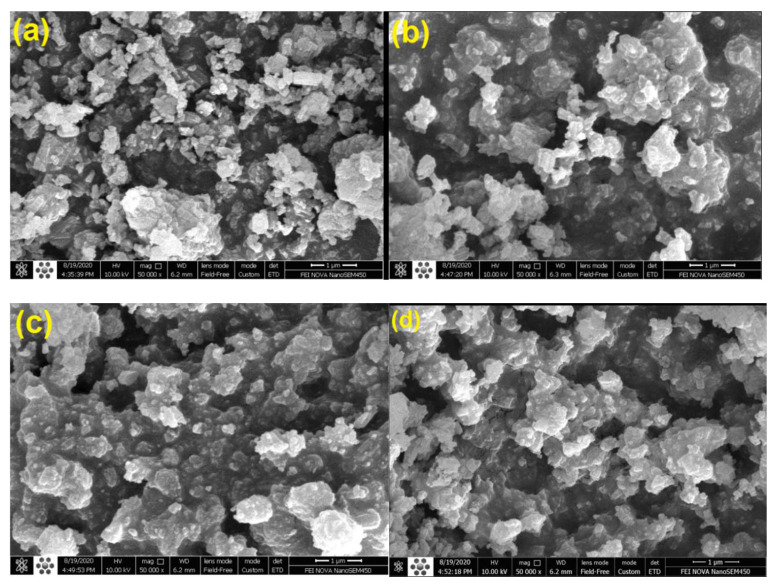
SEM images of HZSM-5 (**a**), 5%Ce/HZSM-5 (**b**), 10%Ce/HZSM-5 (**c**), and 15%Ce/HZSM-5 (**d**).

Figure 7 displays the surface morphology of the parent HZSM-5 zeolites and the Cerium-modified HZSM-5 zeolites catalysts with varying loading weight percent of Cerium (5, 10, and 15%). As can be seen in Figure 7, all of the samples include nanometer-scale, well-crystalline particles that aggregate. Images also show that desilication fractures some of the particles into smaller pieces [83,84]. Images of Cerium-modified zeolites reveal similar pieces, and the addition of rare earth metals does not seem to significantly alter the zeolites’ fundamental structure [33]. Both the unmodified and the Cerium-modified HZSM-5 zeolite catalysts have the same crystallite morphologies and well-organized structure. As a result of the interconnection of tiny particles during the calcination process, the surface of Cerium-modified zeolite catalysts agglomerates; some small particles are covered on the surface after the modification of Cerium metal, and these doped samples seem a little rougher than the pure HZSM-5 [21,33]. 

### 3.2. Catalytic Deoxygenation of the HO over the Parent HZSM-5, 5%La/HZSM-5, 10%La/HZSM-5, and 15%La/HZSM-5 Catalysts

All experiments were performed at 300 °C for 6 h under the same operating conditions in the batch reactor (temperature, time, initial nitrogen pressure, the catalyst to the algal HO ratio (wt. %), and stirring) to compare the conversion and product composition impacts of all manufactured catalysts used in this investigation. Previous research using various reactants served as a foundation for the selection of the operating parameters used in the present investigation of catalytic deoxygenation [8,24,85,86,87].

#### 3.2.1. Conversion of the Algal (HO)

**Figure 8:**
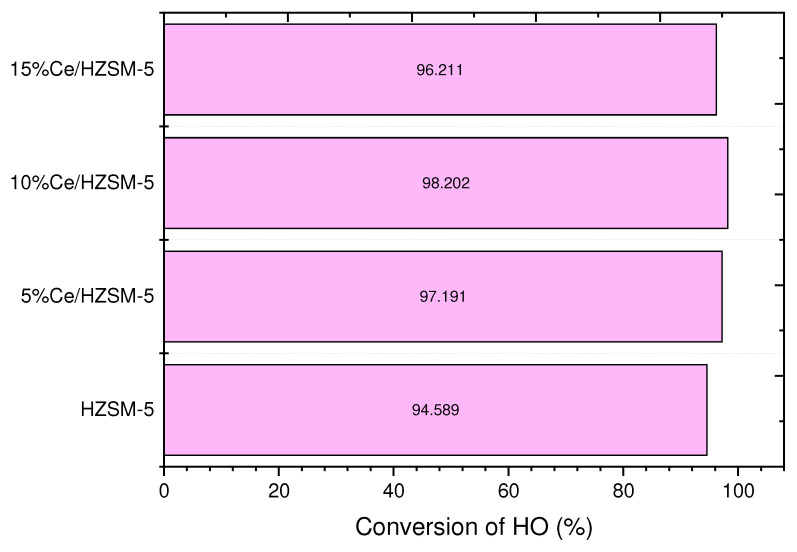
Conversions of the algal (HO) of the catalytic deoxygenation reactions over the parent HZSM-5, 5%Ce/HZSM-5, 10%Ce/HZSM-5, and 15%Ce/HZSM-5 at operating conditions of (batch reactor, 300 °C, 1000 rpm, 7 bar of N_2_ inert gas (initial pressure), the catalyst to the algal HO ratio = 15% (wt. %), and 6 h).

Figure 8 and Table 4 demonstrate that among all the liquid products of the reactions performed in this investigation, the parent HZSM-5 catalyst converted the algal HO to the least extent (94.589%). This poor conversion may be due to the HZSM-5 catalyst’s reduced efficiency under these reaction conditions. In comparison to the liquid product made with the parent HZSM-5, the percentage of algal HO conversion was higher in the liquid product made with Cerium-modified zeolite. Liquid products of catalytic deoxygenation of algae over 5%Ce/HZSM-5, 10%Ce/HZSM-5, and 15%Ce/HZSM-5 had conversion efficiencies of 97.191%, 98.202%, and 96.211%, respectively. In conclusion, the acid sites required to increase algal HO conversion were boosted by the loading of Cerium into HZSM-5 zeolite at various loading percentages.

This explanation might be corroborated by the results of additional investigations. In the meantime, to the best of our knowledge, there is no study in literature similar to the catalytic deoxygenation of the algal HO using these catalysts that were used in this study. The study’s operating parameters (including reactor type, temperature, reaction time, fatty acids or FAMEs reactants, and the starting pressure of the pumped gas) will be compared to those of other similar catalytic investigations.

Researchers Tonya et al. examined the catalytic deoxygenation of soybean oil over Pt/C, Pd/C, and Ni/C in the presence of nitrogen gas, reporting that the percentage of conversion from soybean oil to hydrocarbons under the same working circumstances varies on the kind of catalyst [85].

Mathias et al. studied the catalytic deoxygenation of stearic acid over a range of 20 catalysts at 300 °C and 6 bar of Helium, finding that the percentage of stearic acid converted varies depending on the type of catalyst used. The highest conversion rate was achieved with Pd/C, while the lowest was achieved with Ir/SiO_2_ [86].

As shown by Botas et al. [24], catalytic cracking of rapeseed oil over HZSM-5, Ni/HZSM-5, and Mo/HZSM-5 under identical circumstances yields varying percentages of converted rapeseed oil depending on the oil’s chemical concentration.

Overall, adding Cerium to nanocrystalline HZSM-5 zeolite modifies its acid sites and textural features in significant ways. Cerium oxide is dispersed throughout the zeolite matrix, mostly occupying the micropores. Catalytic performance is also strongly affected by the changes generated in the catalyst characteristics by the metal inclusion.

#### 3.2.2. Chemical Composition Group

**Figure 9:**
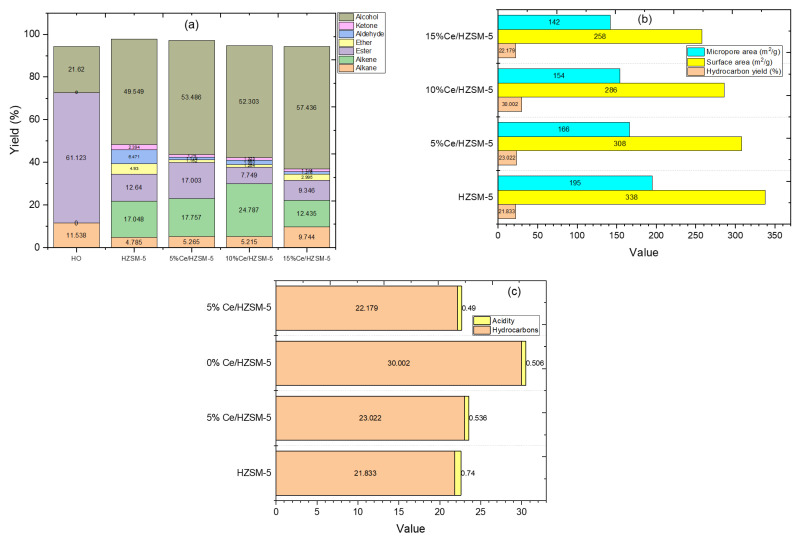
(**a**) The chemical composition groups of the algal HO, and the liquid products from the catalytic deoxygenation of the algal HO over the parent HZSM-5 zeolite and Cerium-modified zeolite with different loading weight percent (batch reactor, 300 °C, 1000 rpm, 7 bar N_2_, catalyst to HO ratio = 15% (wt. %) and 6 h), (**b**) hydrocarbons yield percentages distribution with the surface area and micropore area of the synthesized catalysts, and (**c**) hydrocarbons yield percentages distribution with the acidity of the synthesized catalysts.

Figure 9a and Table 5 show the results of a gas chromatography–mass spectrometry (GC-MS) analysis of the components and contents of algal HO and the products of catalytic deoxygenation of algal HO over the synthesized catalysts in this investigation, respectively. In terms of individual compounds, algal HO has 11.53% alkane, 61.12% esters, and 21.62%; alcohol (phytol). 

The production of the phytol grew in all the experiments undertaken in this work, whereas the yield of alkanes and esters fell and was transformed into other compounds (see GC-MS data of the algal HO and the liquid products of the catalytic deoxygenation in Table 5) (oxygenated compounds and non-oxygenated compounds). In sum, the phytol was not a reactant compound but rather a product compound, while the alkanes with the fatty acid methyl esters were reactant compounds.

This research classified the bio-based chemicals used in the products into seven categories: two for the non-oxygenated compounds (alkanes and alkenes), and five for the oxygenated compounds (esters, ethers, aldehydes, ketones, and alcohols). Figure 9a and Table 5 show the composition groups of the algal HO and the liquid products of the catalytic deoxygenation of the algal HO across all of the synthesized catalysts in this study operating at 300 °C for 6 h with a Catalyst/HO (weight ratio) of 15%, an initial pressure of 7 bar N_2_, and 1000 rpm in a batch reactor.

In terms of the liquid byproducts, the distribution of oxygenated compounds (ester, ether, aldehyde, ketone, and alcohol) was increased compared to the other Cerium-modified zeolite catalysts (12.64%, 4.93%, 6.47%, 2.39%, 49.54%) when the parent HZSM-5 zeolite catalyst was used for the catalytic deoxygenation of the algal HO. Notably, when compared to the products from the other Cerium-modified zeolites catalysts (5%Ce/HZSM-5, 10%Ce/HZSM-5, and 15%Ce/HZSM-5), the parent HZSM-5 catalyst produced a higher percentage of ethers (4.93%) and aldehydes (6.47%), and a lower percentage of the non-oxygenated compounds (hydrocarbons) (21.83%), which were distributed between 4.78% alkane and 17.04% alkene.

In comparison to other Cerium-modified zeolite catalysts (10%Ce/HZSM-5 and 15%Ce/HZSM-5), the 5%Ce/HZSM-5 catalyst generated the highest amount of esters at a rate of 17% and the lowest amount of ether at a rate of 1.16% of the liquid products from catalytic deoxygenation of the algal HO. The percentage of non-oxygenated compounds produced was 23.18%; these were split between 5.26% of alkane and 17.75% of alkene.

The second Cerium-modified zeolite catalyst (10%Ce/HZSM-5) produced the highest percentage of hydrocarbons (30%) among all synthesized catalysts in this study; these hydrocarbons were split evenly between alkanes (5.21%) and alkenes (24.78%). Although this catalyst (10% Ce/HZSM-5) produced 7.74% ester, only 1.25% ether, 1.99% aldehyde, and 1.32% ketone were produced. This catalyst resulted in a 52.3% yield of alcohol.

The third Cerium-modified zeolite catalyst (15%) produced the highest percentage of alcohol (57.43%) compared to the other synthesized catalysts in this study (HZSM-5, 5%Ce/HZSM-5, and 10%Ce/HZSM-5), while the hydrocarbons (alkane and alkene) were produced with the lowest percentage (9.74%, and 12.43%, respectively), compared to the other Cerium-modified zeolite catalysts (5%Ce/HZSM-5 and 10%Ce/HZSM-5). Out of all the Cerium-modified zeolite catalysts, the percentage of ether production was the highest at 2.99%. Utilizing this catalyst (15%Ce/HZSM-5), 1.21% aldehyde was generated. However, this catalyst (15%Ce/HZSM-5) generated the fewest ketones (1.13%) of all of the synthetic catalysts used in this investigation.

This research concluded that the parent HZSM-5 catalyst generated the greatest quantities of oxygenated molecules (ether, aldehyde, and ketone) and the least quantities of hydrocarbons (alkane and alkene). The findings indicated that the least amount of ester was created over a catalyst of 10%Ce/HZSM-5, whereas the greatest quantity of hydrocarbons was synthesized. Alcohol production peaked at 15%Ce/HZSM-5, which is noteworthy.

Therefore, oleochemicals, especially hydrocarbons and alcohol groups, may be generated to manufacture biofuels by catalytic deoxygenation of the algal HO over the parent HZSM-5 zeolite and Cerium-modified zeolite (5%Ce/HZSM-5, 10%Ce/HZSM-5, and 15%Ce/HZSM-5).

Figure 9b shows that there is no definitive link between the physico-chemical characteristics and the yield percentages of the hydrocarbons. The parent HZSM-5 catalyst had the highest micropore and surface areas of all the Cerium-modified zeolite catalysts in this work, but it also had the lowest yield percentages of the hydrocarbon. While (15%Ce/HZSM-5) had the lowest micropore area and surface area, it did not produce the highest hydrocarbon yield percentage (28.25%). Micropore and surface areas were found to decrease from 5% Cerium loading on the parent HZSM-5 to 10%Ce/HZSM-5 to 15%Ce/HZSM-5, as described above (refer to Table 2). In this investigation, the highest hydrocarbons yield (%) (30%) was achieved using 10%Ce/HZSM-5. The physical features of Cerium-loading percentages on the parent HZSM-5 do not seem to be the primary explanation for the onset of hydrocarbons production. Figure 9c shows the correlation between the acidity of all synthesized catalysts and the percentage of hydrocarbons yielded, which is consistent with the findings of Zaki et al., who demonstrated that modifying the parent catalyst (HZSM-5) with some metals (Cu/HZSM-5, Ni/HZSM-5) does not affect its effectiveness for the production of olefins [22]. Hydrocarbon production was lowest for the parent HZSM-5, which also had the highest total acid sites (0.740 mmol/g). A reduction in total acid sites was seen for all of the Cerium-modified zeolite catalysts as compared to the original HZSM-5 (see Figure 5 and Table 3). Total acid sites were 0.536 (mmol/g) for 5%Ce/HZSM-5, 0.506 (mmol/g) for 10%Ce/HZSM-5, and 0.490 (mmol/g) for 15%Ce/HZSM-5 when the Cerium-loading percentage was increased.

The efficiency of catalytic deoxygenation in hydrocarbon formation, however, was shown to vary with varying the proportion of Cerium loaded into the parent HZSM-5.

All of the Cerium-modified zeolites catalysts produced higher hydrocarbon yield percentages than the original HZSM-5 (see Figure 9c). To sum up, as compared to the parent HZSM-5 catalyst, the synthesis of hydrocarbons rose while overall acidity dropped.

Figure 9c demonstrates that the yield percentages of hydrocarbons rise for both 5%Ce/HZSM-5 and 10%Ce/HZSM-5 when the Cerium-loading percentage on the parent HZSM-5 is increased from 5% to 10%.

In contrast, the trend was reversed for 15%Ce/HZSM-5, which has the lowest total acid sites in this study but did not produce the highest yield of the hydrocarbons. This may be because of the high Cerium-loading percentage on the parent HZSM-5 prevented reactants (especially the FAMEs) from reaching the active acid sites of the catalyst, reducing the conversion of the oxygenated compounds of the algal HO into the hydrocarbons. In this investigation, this catalyst resulted in the highest alcohol yield percentages (refer to Table 5).

Other researchers that have explored the impact of metal loading on the zeolite catalysts using a variety of reactants and operating conditions corroborate our findings. Catalytic cracking of swida wilsoniana oil was examined by Li et al., who found a decrease in hydrocarbon yield with increasing metal loading (Cu) on the parent (ZSM-5) above the value of 10% (such as 20%Cu/ZSM-5 and 30%Cu/ZSM-5) and who hypothesized that this was due to the acid sites decreasing as metal loading increased due to the accumulation of the active phase, which inhibited acid site fusing [88]. Zhao et al. investigated the catalytic cracking of camelina oil over Zn/ZSM-5 with varying loading weight percentages of Zn (10, 20, and 30%) and found that Zn/ZSM-5 with a loading percentage of 30 wt. %, Zn reduced the availability of fatty acids for triglyceride breakdown and sped up the deactivation of the catalyst, both of which boosted the production of coke [89].

Researchers Gong et al. looked at the effects of acidity on the activity of a catalyst for the production of propylene by studying the coupling conversion of methanol and a C_4_ alkane over Lanthanum modified HZSM-5 zeolite with varying loading metals (0.5, 1.5, 5, and 7%) La/HZSM-5. They demonstrated that adding Lanthanum to HZSM-5 changed the acidity and reduced the strong acid sites (Bronsted acid sites), leading them to the conclusion that the impact of acidity on the catalyst’s activity in the propylene synthesis process was highly nuanced [31].

#### 3.2.3. The Distribution of Carbon Numbers

**Figure 10:**
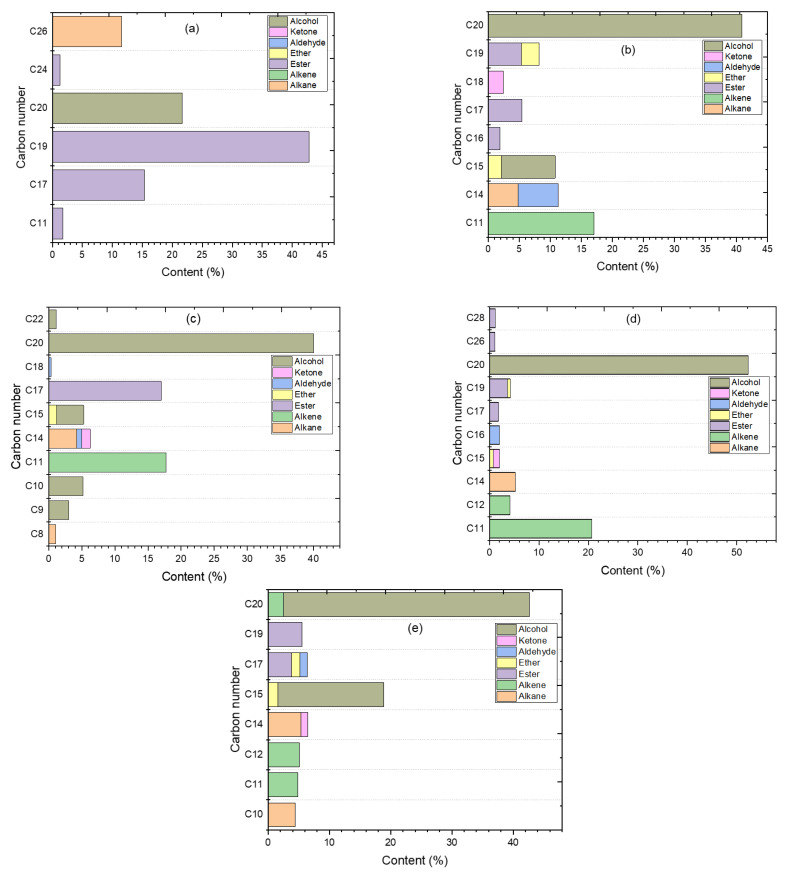
Carbon number distribution for the algal HO (**a**) and the products of the catalytic deoxygenation of the HO over HZSM-5 (**b**), 5%Ce/HZSM-5 (**c**), 10%Ce/HZSM-5 (**d**), and 15%Ce/HZSM-5 (**e**).

Considering the algal HO conversion into product chemical composition groups, the results of Figure 10a and Table 5 indicate that the catalytic deoxygenation over the synthesized catalysts conditions of initial 7 bar N_2_ pressure, 300 °C, 6 h, 15% weight ratio of catalyst/HO, and 1000 rpm highlighted the high conversion of algal HO and revealed the intriguing chemical groups. To compare the carbon number distributions of the liquid products produced under these working circumstances to those of all the synthesized catalysts in this investigation, the data was given in terms of carbon number and product yield.

As previously stated, there were seven categories of liquid products, including two groups for non-oxygenated molecules (alkanes and alkenes) and five groups for oxygenated compounds (esters, ethers, aldehydes, ketones, and alcohols). In addition to non-oxygenated molecules (alkenes) and oxygenated compounds (alcohols), minor quantities of alkanes, esters, ethers, aldehydes, and ketones were also discovered. The components and amounts of algal HO are shown in Figure 10a and Table 5. The primary component of the algal HO is composed of three chemical groups: alkane (11.53%), esters (61.12%), and alcohol (phytol) (21.6%). According to the GC-MS data (Figure 10a and Table 5), the production of phytol rose in all of the trials done for this investigation, whereas the yield of alkanes and esters declined and were transformed into other chemicals (oxygenated compounds and non-oxygenated compounds). In conclusion, phytol was not a reactant compound, but it may be regarded as a product chemical in this research, while alkanes and fatty acid methyl esters can be considered reactants.

As indicated in Figure 10a, the esters were the most abundant components of algal HO (61.12%). They were mostly dispersed in C_19_ carbon atoms (19) with a ratio of 42.776% and were distributed 15.08% in 9,12,15-Octadecatrienoic acid, methyl ester, (Z,Z,Z)- (C_19_H_32_O_2_), and 27.69% in 9,12-Octadecadienoic acid, methyl ester (C_19_H_34_O_2_). The distribution of the remaining esters was as follows: 15.32% Hexadecanoic acid, methyl ester (C_17_), 1.728% 6-Octen-1-ol, 3,7-dimethyl-, formate (C_11_), and 1.294% Di-*n*-octyl phthalate (C_24_). Hexacosane include C_26_ carbon atoms (26) constituting 11.53% of the alkane group (C_26_H_54_). At phytol, the alcohol chemical group was represented by C_20_ carbon atoms in a proportion of 21.62%. (C_20_H_40_O).

For the liquid product of the catalytic deoxygenation of algal HO over the parent HZSM-5 zeolite shown in Figure 10b, the products primarily consisted of the alcohol group (49.54%), which were primarily distributed in C_20_ carbon atoms (20) in the proportion of 40.85% in phytol (C_20_H_40_O) and C_15_ carbon atoms (15) in the proportion of 8.69% in 1-Dodecanol, 3,7,11- (C_15_H_32_O). The other major components were non-oxygenated compounds (hydrocarbons), which were distributed in alkane and alkene. The overall proportion of hydrocarbons was 21.83%, with C_11_ carbon atoms comprising 17.048% of 5-Ethyl-1-nonene (C_11_H_22_) and C_14_ carbon atoms comprising 4.78% of Tetradecane (C_14_H_30_). Low percentages were found for the other chemical groups (esters, ethers, aldehydes, and ketones). The total amount of esters was 12.64%, which was distributed as 5.411% C_17_, 1.88% C_16_, and 5.34% C_19_, which were Hexadecanoic acid, methyl ester (C_17_H_34_O_2_), Carbonic acid, butyl undec-10-enyl ester (C_16_H_30_O_3_), and trans-13-Octadecenoic acid, methyl ester (C_19_H_36_O_2_), respectively. Ethers were discovered in 4.930 % of the compounds, with the highest concentration in C_19_ (1.46% in Disparlure (C_19_H_38_O) and 1.37% in Tetrahydropyran 12-tetradecyn-1-ol ether (C_19_H_34_O_2_)) and C_15_ (Oxirane, tridecyl-) (C_15_H_30_O), respectively. Aldehyde group was 6.47% of (C_14_) in the Tetradecanal product (C_14_H_28_O). At 2.39% in (C_18_) of 2-Pentadecanone, 6,10,14-trimethyl (C_18_H_36_O), the ketone group was detected in the product. 

For the liquid product of the catalytic deoxygenation of algal HO over 5%Ce/HZSM-5 zeolite, Figure 10c, the products primarily consisted of non-oxygenated compounds (hydrocarbons) with a total percentage of 23.02%, which primarily distributed in the alkene group with (C_11_) carbon atom of 17.75%, which was 5-Ethyl-1-nonene (C_11_H_22_); and in alkane group with (C_14_) that was 4.2% of Tetradecane (C_14_H_30_) and C_8_ in 1.05% of Octane (C_8_H_18_). The second principal product of this catalyst (5%Ce/HZSM-5) is the alcohols group, with a proportion of 53.48%, mostly distributed in (C_20_) carbon atoms of 40.03% Phytol (C_20_H_40_O); (C_15_) in 4.11% of 1-Dodecanol, 3,7,11-trimethyl- (C_15_H_32_O); (C_9_) in 2.99% of 2-Propylcyclohexanol (C_9_H_18_O), (C_10_) in 5.2% of 2-Norpinanol, 3,6,6-trimethyl- (C_10_H_18_O), and (C_22_) in 1.13% of Behenic alcohol (C_22_H_46_O). 17% of the dispersed product (C_17_) included the esters group; 2.8% and 14.19% of Hexadecanoic acid, methyl ester (C_17_H_34_O_2_), and 2-(Prop-2-enyloxy)tetradecane (C_17_H_32_O_2_), respectively. The ether group was 1.16% of the tridecyl-oxyirane (C_15_) (C_15_H_30_O). The aldehyde group comprised 1.17% of the liquid product, distributed as 0.79% (C_14_) of Tetradecanal (C_14_H_28_O) and 0.38% (C_18_) of 13-Octadecenal, (Z)- (C_18_H_34_O). The ketone group comprised 1.29% of 9-(Tetrahydropyran-2-yloxy)-4,6-dioxatricyclo[5.3.1.0(3,21ndecanecan-5-one in C_14_ (C_14_H_20_O_5_).

For the liquid product of the catalytic deoxygenation of algal HO over 10% Ce/HZSM-5 zeolite, as shown in Figure 10d, the products primarily consisted of non-oxygenated compounds (hydrocarbons) with a total percentage of 30%, which predominantly distributed in the alkene group with (C_11_) carbon atom of 20.65% that was 5-Ethyl-1-nonene (C_11_H_22_); and 4.13% in (C_12_) of 1-Undecene, 8-methyl- (C_12_H_24_) (C_14_), while the alkane group with 5.21% in (C_14_) that was Tetradecane (C_14_H_30_). The alcohol group is the second major product of this catalyst (10%Ce/HZSM-5) with a proportion of 52.3% in (C_20_) carbon atom of 48% Phytol (C_20_H_40_O) and 4.29% of 3,7,11,15-Tetramethyl-2-hexadecen-1-ol (C_20_H_40_O). The esters group accounted for 7.74% of the product and was comprised of 1.79% (C_17_), 1.1% (C_26_), 1.19% (C_28_), and 3.65% (C_19_) of Hexadecanoic acid, methyl ester (C_17_H_34_O_2_); Oxalic acid, hexyl octadecyl ester (C_26_H_50_O_4_); Decyl oleate (C_28_H_54_O_2_). The ether group was identified in the product at a concentration of 1.254%; it was spread between 0.53% (C_19_) and 0.71% (C_15_) of Disparlure (C_19_H_38_O) and Oxirane, tridecyl- (C_15_H_30_O), respectively. A total of 1.99% of cis-9-Hexadecenal (C_16_) belonged to the aldehyde group in the liquid product (C_16_H_30_O). The ketone group comprised 1.32% of 4,7,7-Trimethyl-5-(tetrahydropyran-2-yloxy)-bicyclo[2.2.1]heptan-2-one (C_15_) (C_15_H_24_O_3_).

For the liquid product of the catalytic deoxygenation of algal HO over 15%Ce/HZSM-5 zeolite, Figure 10e shows that the products primarily consisted of the alcohols group (57.43%), with (C_20_) carbon atoms (20) in phytol (C_20_H_40_O) accounting for 40.14% and (C_15_) carbon atoms (15) in 1-Dodecanol, 3,7,11-trimethyl- (C_15_H_32_O) accounting for 17.28%. The remaining 22.17% of the sample consisted of non-oxygenated compounds (hydrocarbons), which were mostly distributed as follows: in alkene 4.83% (C_11_), 5.1% (C_12_), 2.49% (C_20_), and alkane 5.36% (C_14_) with 4.38% (C_10_) of alkanes those were 5-Ethyl-1-nonene (C_11_H_22_), 1-Undecene, 8-methyl- (C_12_H_24_), 2-Hexadecene, 2,6,10,14-tetramethyl- (C_20_H_40_), Tetradecane (C_14_H_30_), and Bicyclo[3.1.1]heptane, 2,6,6-trimethyl-, (1.alpha.,2.beta.,5.alpha.), respectively. Hexadecanoic acid, methyl ester (C_17_H_34_O_2_), and 11-Octadecenoic acid, methyl ester (C_19_H_36_O_2_) constituted 3.78% (C_17_) and 5.55% (C_19_) of the total quantity of esters, respectively. Ethers group was identified in 2.995 percent of those mostly dispersed in 1.6% (C_15_) of Oxirane, tridecyl- (C_15_H_30_O), and 1.38% (C_17_) of 2H-Pyran, 2-(7-dodecyloxy)tetrahydro-(C_17_H_30_O_2_). The overall percentage of the Aldehyde group in 2-Heptadecenal (C_17_) was 1.21%. (C_17_H_32_O). A total of 1.13% of (C_14_) of 1-Cyclohexene, 1,3,3-trimethyl-2-(1-methylbut-1-en-3-on-1-yl)- (C_14_H_22_O) had a ketone group.

#### 3.2.4. Outstanding Bio-Based Chemical Products

The catalytic deoxygenation of algal HO over the parent HZSM-5 catalyst and cerium-modified zeolites catalysts was evaluated using Equation (1), and the percentage yield of the selected outstanding hydrocarbons (alkanes and alkenes) and alcohol groups is shown in Table 6.

In Table 6, we can see that the non-oxygenated compounds (hydrocarbons) and oxygenated compounds (alcohols) had greater yield percentages (20%) during the catalytic deoxygenation processes of the algal HO utilizing the native HZSM-5 and Cerium-modified zeolites catalysts. So, these catalysts are appealing for catalytic deoxygenation processes of FAMEs, even at low nitrogen pressure (7 bar), which means cheaper costs.

**Figure 11:**
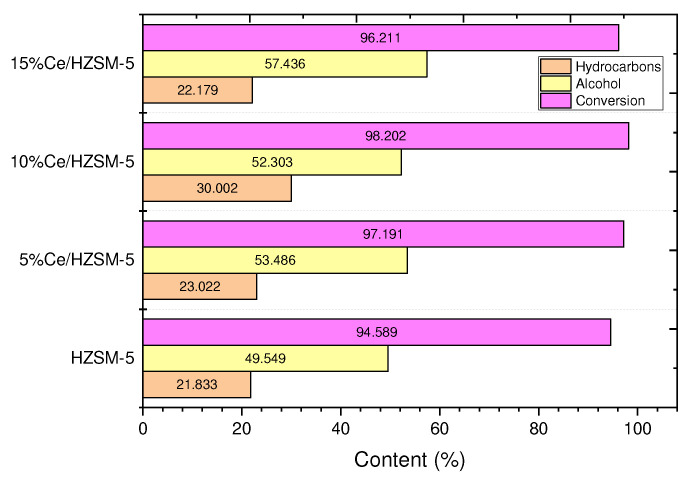
The conversion percentage of the algal HO and the yield percentages of the outstanding chemicals of the hydrocarbons and alcohol from the catalytic deoxygenation of the algal HO over the parent HZSM-5 zeolite and Cerium-modified HZSM-5 zeolite with different loading weight percentage (batch reactor, 300 °C, 1000 rpm, 7 bar N_2_ initial gas (inert gas), the catalyst to algal HO ratio = 15% (wt. %) and 6 h).

The yields of hydrocarbons obtained from the catalytic deoxygenation of algal HO using all of the Cerium-modified zeolites catalysts were greater than the yields of hydrocarbons obtained using the original HZSM-5 catalyst, as shown in Table 6 and Figure 11.

Hydrocarbon yields from catalytic deoxygenation of algal HO were 21.83%, 23.02%, 30%, and 22.17% for the parent HZSM-5, 5%Ce/HZSM-5, 10%Ce/HZSM-5, and 15%Ce/HZSM-5, respectively. In this work, catalytic deoxygenation of algal HO in the batch reactor at 300 °C for 6 h under 7 bars of initial inert N_2_ gas, with a catalyst to HO ratio of 15% (wt. %), resulted in the greatest yield value for the produced hydrocarbons (30%) and the highest conversion percentage (98.2%).

Furthermore, the algal HO in this investigation had a lower conversion percentage (94.58%) and yield of generated hydrocarbons (21.83%) over the parent HZSM-5. One possible explanation is that current levels of other chemical substances contain oxygen. The formation of aldehydes, ketones, and ethers rather than hydrocarbons during catalytic deoxygenation of algal HO may account for the observed reduction in hydrocarbons compared to the parent HZSM-5 catalyst (refer to Table 5).

It is possible that the low quantity of hydrogen created during catalytic deoxygenation of the algal HO was not adequate to saturate the double bonds during these reactions, which would explain why the yield percentages of alkanes are substantially lower than those of alkenes in this work. As was previously indicated, however, this research was carried out using the inert gas of nitrogen as the initial pressure.

In conclusion, Cerium has a major impact on the overall acid sites, notably the acidic Bronsted sites, when added to the parent HZSM-5 zeolite. As was mentioned in the section on Ammonia TPD, this area of strong acid sites (the Bronsted sites) is thought to be the major catalytic core and functions as dominating acid sites during catalytic deoxygenation processes [19].

This study’s operating conditions (reactor type, time, temperature, and the initial value of the charged gas pressure) are very close to those in the studies shown in Table 7, which shows the results of other studies relating to the catalytic deoxygenation process using different types of catalysts for producing hydrocarbons from different feeds (fatty acids or FAMEs). Our findings, however, show that Cerium-modified zeolite exhibited deoxygenating activity in the presence of a relatively inexpensive inert gas (nitrogen), suggesting that the production of hydrocarbons could be accomplished without the use of hydrogen gas, thereby significantly reducing the process’s cost.

The catalytic cracking deoxygenation with various catalysts and reactants under starting H_2_ pressure or N_2_ inert gas pressure has been the subject of many scientific discussions. Compared to research into catalytic deoxygenation using hydrogen gas as the initial pressure, research into the process using inert gas as the initial pressure is much lower. Additionally, the catalytic deoxygenation of algal HO utilizing the catalysts used here is unique to this work. So, this research will compare its operating conditions (such as reactor type, temperature, duration, fatty acids or FAMEs reactants, and the starting pressure of the pumped gas) with those of other similar catalytic investigations (refer to Table 7).

Focusing on the catalytic deoxygenation under H_2_ gas pressure. Catalytic deoxygenation of palm kernel oil and hydrolysis of palm kernel oil over HBeta zeolite under 10 bar of H_2_ as starting pressure resulted in 82 ± 3%, and 24 ± 9% yields of hydrocarbons, respectively, as stated by Sousa et al. (see Table 7) [8]. When comparing the yields of hydrocarbons produced by catalytic deoxygenation of olein oil and hydrolyzed olein oil using the same catalyst and the same operating conditions, as shown in Table 7, the hydrocarbons’ yield percentages were 43 ± 3%, and 98 ± 4%. They concluded that the type of reactants (the volume of reactant molecule and the length of carbon chain of the reactants) greatly affects the amount of hydrocarbons that are produced.

When utilizing a Pd/C catalyst and an initial pressure of 25 bar of H_2_, Meller et al. investigated how changing the solvent and temperature affected the catalytic deoxygenation, which led to the formation of hydrocarbons from hydrolyzed castor oil (refer to Table 7). In their discussion of the relationship between solvent type and hydrocarbon yield [9], provided in Table 7, they emphasize the importance of reaction temperature.

At 260 °C for 8 h, the overall yield of the produced hydrocarbons was about 56% after catalytic deoxygenation of stearic acid over 10%Ni/HZSM-5 at 40 bar of starting H_2_ pressure [10] (refer to Table 7). Peng et al. also investigated the catalytic conversion of microalgae oil over 10%Ni/ZrO_2_ at 270 °C, 40 bar of initial H_2_ pressure, and in the absence of solvent, and they discussed the effect of reaction time on the total yield of the hydrocarbons under the same operating conditions, with the total yield of the hydrocarbons at 6 h being 72% and at 4 h being 61%, respectively [11], (as presented in Table 7). Under 30 bar of starting H_2_ pressure, 300 °C, and 5 h, the maximum yield of the hydrocarbons was 12.750 % when utilizing hexane as a solvent, as reported by Duongbia et al. [12], (refer to Table 7). This article focuses on catalytic deoxygenation utilizing inert gas pressure. At the same catalyst, temperature, feed/catalyst ratio, reaction time, and selectivity of the hydrocarbons, Snare et al. [13] found that the initial gas pressure had a significant effect on the conversion percentage and the amount of hydrocarbons produced. A study by Morgan et al. [90] on catalytic deoxygenation of soybean oil using 20%Ni/Al_2_O_3_ under 7 bar of inert gas (N_2_) at 350 °C showed a maximum hydrocarbon production percentage of 79.5% and a conversion of 74% (refer to Table 7). Catalytic deoxygenation of stearic acid over Pd/Al_2_O_3_ at 350 °C, 7 bars of nitrogen inert gas pressure, and 6 h yielded a 43% conversion rate, as shown in Table 7 by Hollak et al. [91].

Overall, in this study, the catalytic deoxygenation of the algal HO over the parent HZSM-5 produced higher amounts of non-oxygenated compounds in comparison with the Cerium-modified zeolites. This observation is in line with Li et al. [92]. They used HZSM-5 and 5%Fe/HZSM-5 in the catalyzed liquefaction of cellulose in presence of the solvent (*n*-heptane) at 350 °C in a batch reactor, and they observed that the parent HZSM-5 gave higher yields of oxygenated compounds and lower yields of non-oxygenated compounds compared with 5%Fe/HZSM-5.

According to the aforementioned studies, the yield percentage of the hydrocarbons produced from the catalytic deoxygenation is influenced by many variables, including temperature, solvent use, solvent type, reactant type, feed/catalyst ratio, reaction time, and the initial pressure pumped into the reactor before the reaction takes place.

Specifically addressing the alcohol molecules that were generated, during catalytic deoxygenation reactions of the algal HO, all of the Cerium-modified zeolite catalysts produced more alcohol than the parent HZSM-5 catalyst, according to Table 8 and Figure 11. In particular, the alcohol output from catalytic deoxygenation of algal HO was 49.549% for the parent HZSM-5, 53.48% for 5%Ce/HZSM-5, 52.30% for 10%Ce/HZSM-5, and 57.43% for 15%Ce/HZSM-5. The results of catalytic deoxygenation of algal HO in a batch reactor at 300 °C, 6 h, and 7 bar of initial inert N_2_ gas, the catalyst to the algal HO ratio = 15% (wt. %) showed a higher yield value for the obtained alcohols (50.943%), despite a lower conversion percentage (94.589%) when compared to the parent HZSM-5 (refer to Table 6, and Figure 11).

Overall, the yield percentage of hydrocarbons and alcohols was lowest for the parent HZSM-5 catalyst. Furthermore, the maximum yield percentage of hydrocarbons was obtained from 10%Ce/HZSM-5. One possible explanation is that HZSM-5 has more strong acid sites (0.214 mmol/g) than 10% Ce/HZSM-5 (0.166 mmol/g). Additionally, strong acid sites play a crucial role as the primary catalytic center in the catalytic deoxygenation of oxygenated substances [19]. Therefore, a reduced number of strong acid sites may improve oxygenate-to-hydrocarbon upgrading. Over 15%Ce/HZSM-5, the alcohol production increased significantly.

The following is an analysis of the catalytic deoxygenation process, which includes a discussion of the creation of oxygenated molecules such as alcohol. The majority of catalytic deoxygenation-related research discusses the yields of non-oxygenated compounds (hydrocarbons), whereas the minority of studies address the generation of oxygenated compounds in catalytic deoxygenation (hydrocarbons). Table 8 displays the results of investigations on catalytic deoxygenation and the yield percentages of alcohol compounds achieved under different operating settings with different reactants and different catalysts. However, as was indicated before, no prior research has attempted catalytic deoxygenation of algal HO using the catalysts used here. Thus, investigations of near catalytic cracking will be compared concerning the reactants and the percentage yield of the generated alcohol.

According to the study by Duongobia et al. [12], which examined the impact of reaction duration on the yield percentage of alcohol during catalytic hydrotreating of palmitic acid over Limonite catalyst, the yield percentage of alcohol was 51.84% after 5 h and 38.35% after 3 h (refer to Table 8).

Under the same operating conditions (refer to Table 8), when J. Li et al. studied the catalyzed liquefaction of cellulose assisted by glycerol over HZSM-5 and 5%Fe/HZSM-5, they discovered that the Fe metal modification of the parent HZSM-5 resulted in a lower yield percentage of alcohol (20%) than the parent HZSM-5 (26%) [93].

The catalytic deoxygenation of Lauric acid over 5% Pd/C was investigated by Rozmyslowics et al. under identical circumstances (except for the kind of starting pressure gas), and the researchers found that the percentage of alcohol output was significantly influenced by the type of initial pressure (refer to Table 8). Catalytic deoxygenation of stearic acid over 4% Ru/TiO_2_ yielded 20% alcohol at 1 h but none at 6 h, as shown by Rozmyslowics et al. [93] (Table 8), using identical working conditions (apart from reaction duration). Two experiments were conducted under the same operating conditions (except for the catalyst type) (Table 8), and the total yield percentage of alcohol was 81% for the products from 4% Re/TiO_2_, while the products from 4% Ru/TiO_2_ contained no alcohol [93]. This demonstrated that the type of catalyst has a significant effect on the yield percentage of alcohol for the catalytic deoxygenation of stearic acid.

In a fixed bed reactor, Zheng et al. [94] found that the composite catalyst (35% Al_2_O_3_/CaO) produced the greatest yield percentage of alcohol at 12.3% from the catalytic cracking of soybean oil (refer to Table 8).

Balasundram et al. [21] reported that the alcohol output percentage in the catalytic pyrolysis of Sugarcane bagasse was around 14% over the parent HZSM-5, and 5% above 1%Ce/HZSM-5.

Based on the researches presented above, it can be concluded that the yield percentage of the alcohols produced from various reactions, such as catalytic deoxygenation, is affected by several variables, such as temperature, catalyst type, reaction time, and the initial pressure pumped into the reactor before the reaction takes place.

#### 3.2.5. Liquid Product Characterization

Catalytic deoxygenation of algal HO over parent HZSM-5 and Cerium-modified zeolite catalysts yielded the same liquid products when run at 300 °C for 6 h with a Catalyst/HO (weight ratio) of 15%, at 1000 rpm, in a batch reactor pressurized with 7 bar N_2_. Calculated elemental compositions of liquid products are shown in Table 9 using Equation (3).

Carbon and hydrogen weight percentages were rising, whereas oxygen weight percentages were dropping, in the products of catalytic deoxygenation from 5%Ce/HZSM-5, 10%Ce/HZSM-5, and 15%Ce/HZSM-5 when compared to the algal HO. The parent HZSM-5 catalyst showed a rise in carbon and a fall in hydrogen and oxygen. Similar to the Jafarian research [95], as indicated in Table 9, the HHV of the catalytic deoxygenation liquid products over the parent HZSM-5, 5%Ce/HZSM-5, 10%Ce/HZSM-5, and 15%Ce/HZSM-5 was raised by 33.23, 33.48, 34.05, and 33.82 MJ/kg, respectively, when compared to the HHV (MJ/kg) of the algal HO (32.37). The degree of deoxygenation percentage (DOD%) of the liquid products from the modified Cerium zeolite (10%Ce/HZSM-5) was higher than the DODs of the liquid products from other synthetic catalysts in this study, as calculated from the O/C molar ratios using Equation (3). The absence of oxygen may improve the fuel characteristics, such as viscosity and acidity, of the products [96]. In contrast to fossil crude oil, the HHVs of the products of all catalysts were low [97]. 

**Figure 12:**
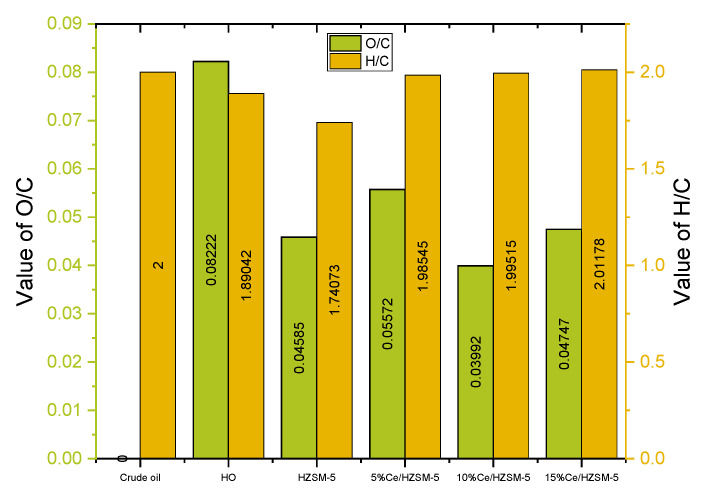
Van Krevelen diagram of the liquid products produced by catalytic deoxygenation of the algal HO over HZSM-5 and Cerium-modified zeolite catalysts.

Table 9 provides a visual representation of the atomic ratios of hydrogen to carbon and oxygen to carbon in the form of a Van Krevelen diagram (Figure 12). An increased H/C ratio and a decreased O/C ratio were observed in the liquid products of all Cerium-modified zeolite catalysts as compared to the algal HO. Both the H/C and O/C atomic ratios of the parent HZSM-5 zeolite decreased as compared to the raw algal HO. The maximum H/C ratio for Cerium-modified zeolites was 2.01 at 15%Ce/HZSM-5 and the minimum O/C ratio was 0.03 at 10%Ce/HZSM-5. While the liquid products’ H/C ratios were far higher than those of fossil crude oil, the O/C ratios remained high compared to fossil crude oil. Note that the highest DOD% was 51.44% over 10%Ce/HZSM-5 (see Table 9), which is low compared to the amount of hydrogen used. This result was compared to the result of a previous study that involved the hydrotreating of palmitic acid over Ni/LY catalyst under 30 bar H_2_ at 300 °C in a batch reactor, where the highest DOD% was 65.15%.

## 4. Conclusions

For catalytic conversion of the algal HO to non-oxygenated compounds and oxygenated compounds, the performance of the parent HZSM-5 zeolite catalyst and Cerium (5%, 10%, and 15%) -impregnated HZSM-5 has been tested in a batch reactor. The proportion of cerium loading was the causal factor in the observed morphological and textural changes. In general, physical qualities cannot be used to infer any kind of link. Multiple percentages of Cerium added into HZSM-5 zeolite improved acid sites essential for algal HO conversion. While the conversion of algal HO using the original HZSM-5 was 94.58 percent, employing all Cerium-modified HZSM-5 resulted in conversion percentages in the range of 96.21 to 98.2%. Incorporating Cerium into HZSM-5 provides significant effects, including a boost in hydrocarbon output (22.17–30%). Generally, the increasing performance of catalysts on upgrading algal HO into hydrocarbons is in the following order: 10%Ce/HZSM-5 > 5%Ce/HZSM-5 > 15%Ce/HZSM-5 > HZSM-5. Alcohol products from Cerium-modified zeolite ranged from 52.3 to 57.43%, whereas those from the original HZSM-5 were only 49.54%. Liquid products from Cerium-modified HZSM-5 catalysts had DODs in the range of 32.22–51.44%, while liquid products from the parent HZSM-5 catalyst had a DOD of 44.23%. Furthermore, the HHV of liquid products from all Cerium-modified HZSM-5 catalysts was higher than that of liquid products from the parent HZSM-5 (33.23 MJ/Kg). 10%Ce/HZSM-5 had the greatest conversion of the algal HO, the yield of hydrocarbons, HHV, and DOD% (98.2%, 30%, 34.05 MJ/Kg, and 51.44%, respectively) among all the synthesized catalysts in this investigation.

## Figures and Tables

**Table 1:** The relative crystallinity for parent HZSM-5 and Cerium-modified zeolites catalysts.

**No.**	**Catalyst Name**	**Relative Crystallinity (%)**
1	HZSM-5	100
2	5%Ce/HZSM-5	115
3	10%Ce/HZSM-5	78
4	15%Ce/HZSM-5	57

**Table 2:** Texture properties of the parent HZSM-5 and Cerium-modified HZSM-5 with different loading weight percentages modified HZSM-5.

No	Catalyst	S_BET_ (m^2^/g)	**S_micro_** **(m^2^/g)**	**S_extern_** **(m^2^/g)**	**V_total_** **(cm^3^/g)**	**V_micro_** **(cm^3^/g)**	Average Particle Size (nm)
1	HZSM-5	338	195	143	0.22	0.100	17
2	5%Ce/HZSM-5	308	166	142	0.2	0.085	19
3	10%Ce/HZSM-5	286	154	131	0.19	0.081	20
4	15%Ce/HZSM-5	258	142	115	0.17	0.074	23

S_BET_: BET surface area was calculated by Brumauer–Emmett–Teller (BET) mode. S_micro_: Micropore area was determined from the t-Plot micropore area. S_extern_: External surface area was determined from the t-Plot area. V_total_: The total pore volumes were obtained from the adsorbed amount at P/P_0_ = 0.95. V_micro_: The micropore volume was measured by the t-plot method.

**Table 3:** NH_3_-TPD properties of HZSM-5, 5%Ce/HZSM-5, 10%Ce/HZSM-5, and 15%Ce/HZSM-5.

Catalyst	Low Peak Temperature Point (°C) (Weak Acid Peak)			High Peak Temperature Point (°C) (Strong Acid Peak)			Total Acid Amount (Total NH_3_ Amount mmol/g)
	T (°C)	TCD (V)	NH_3_ Amount (mmol/g)	T (°C)	TCD (V)	NH_3_ Amount (mmol/g)	
**HZSM-5**	216	0.0808	0.526	439	0.0295	0.214	0.740
**5%Ce** **/HZSM-5**	208.500	0.0523	0.358	449	0.0221	0.178	0.536
**10%Ce** **/HZSM-5**	214	0.0459	0.340	420	0.0218	0.166	0.506
**15%Ce** **/HZSM-5**	212.900	0.0394	0.331	412.3	0.0201	0.159	0.490

**Table 4:** Mass balances (wt. %) of the algal HO compound in the feed, and the compounds of liquid products for the conversion of algal HO over HZSM-5, 5%Ce/HZSM-5, 10% Ce/HZSM-5, and 15%Ce/HZSM-5.

Compounds of the Algal (HO)	Molecular Formula	Content of the Compound in the Feed (HO) (wt. %)	Content (wt. %) of the Compound in the Liquid Product of the Catalytic Deoxygenation Reactions for the Algal HO as a Function of the Cerium-loading Percentage on the Parent HZSM-5			
			HZSM-5	5%Ce/HZSM-5	10%Ce/HZSM-5	15%Ce/HZSM-5
Hexacosane	C_26_H_54_	11.53	0	0	0	0
6-Octen-1-ol, 3,7-dimethyl-, formate	C_11_H_20_O_2_	1.72	0	0	0	0
9,12,15-Octadecatrienoic acid, methyl ester, (Z,Z,Z)-	C_19_H_32_O_2_	15.08	0	0	0	0
Hexadecanoic acid, methyl ester	C_17_H_34_O_2_	15.32	5.41	2.8	1.79	3.78
9,12-Octadecadienoic acid, methyl ester	C_19_H_34_O_2_	27.69	0	0	0	0
Di-*n*-octyl phthalate	C_24_H_38_O_4_	1.29	0	0	0	0
Phytol	C_20_H_40_O	21.62	40.85	40.03	48	40.14
others	-	5.71	0	0	0	0
**Conversion (%) of the algal HO in the catalytic deoxygenation reactions as a function of the Cerium-loading percentage on the parent HZSM-5**			**94.58**	**97.19**	**98.20**	**96.21**

**Table 5:** The main components and content of the algal HO and the products of the catalytic deoxygenation reactions for the algal HO over the parent HZSM-5 zeolite and Cerium-modified HZSM-5 zeolite with different loading weight percent (batch reactor, 300 °C, 1000 rpm, 7 bar N_2_ inert gas (initial pressure), the catalyst to algal HO ratio = 15% (wt. %), and 6 h).

Compound	Molecular Formula	Algal Hydrolyzed Oil (HO)	HZSM-5	5%Ce/ HZSM-5	10%Ce/ HZSM-5	15%Ce/ HZSM-5
ALKANE						
Hexacosane	C_26_H_54_	11.53				
Tetradecane	C_14_H_30_		4.78	4.2	5.21	5.36
Octane	C_8_H_18_			1.05		
Bicyclo[3.1.1]heptane, 2,6,6-trimethyl-, (1.alpha.,2.beta.,5.alpha.)	C_10_H_18_					4.38
**TOTAL ALKANES**		**11.53**	**4.78**	**5.26**	**5.21**	**9.74**
**ALKENS**						
5-Ethyl-1-nonene	C_11_H_22_		17.04	17.75	20.65	4.83
1-Undecene, 8-methyl-	C_12_H_24_				4.13	5.1
2-Hexadecene, 2,6,10,14-tetramethyl-	C_20_H_40_					2.49
**TOTAL ALKENS**		**0**	**17.04**	**17.75**	**24.78**	**12.43**
**ESTERS**						
6-Octen-1-ol, 3,7-dimethyl-, formate	C_11_H_20_O_2_	1.72				
9,12,15-Octadecatrienoic acid, methyl ester, (Z,Z,Z)-	C_19_H_32_O_2_	15.08				
Hexadecanoic acid, methyl ester	C_17_H_34_O_2_	15.32	5.41	2.8	1.79	3.78
Carbonic acid, butyl undec-10-enyl ester	C_16_H_30_O_3_		1.88			
9,12-Octadecadienoic acid, methyl ester	C_19_H_34_O_2_	27.69				
Di-*n*-octyl phthalate	C_24_H_38_O_4_	1.29				
trans-13-Octadecenoic acid, methyl ester	C_19_H_36_O_2_		5.34			
2-(Prop-2-enoyloxy)tetradecane	C_17_H_32_O_2_			14.19		
Oxalic acid, hexyl octadecyl ester	C_26_H_50_O_4_				1.1	
Decyl oleate	C_28_H_54_O_2_				1.19	
6-Octadecenoic acid, methyl ester, (Z)-	C_19_H_36_O_2_				3.65	
11-Octadecenoic acid, methyl ester	C_19_H_36_O_2_					5.55
**TOTAL ESTERS**		**61.12**	**12.64**	**17**	**7.74**	**9.34**
**ETHERS**						
Disparlure	C_19_H_38_O		1.46		0.53	
Tetrahydropyran 12-tetradecyn-1-ol ether	C_19_H_34_O_2_		1.37			
Oxirane, tridecyl-	C_15_H_30_O		2.08	1.16	0.71	1.6
2H-Pyran, 2-(7-dodecynyloxy)tetrahydro-	C_17_H_30_O_2_					1.38
**TOTAL ETHERS**		**0**	**4.93**	**1.162**	**1.25**	**2.99**
**ALDEHYDES**						
Tetradecanal	C_14_H_28_O		6.47	0.79		
13-Octadecenal, (Z)-	C_18_H_34_O			0.38		
cis-9-Hexadecenal	C_16_H_30_O				1.99	
2-Heptadecenal	C_17_H_32_O					1.21
**TOTAL ALDEHYDES**		**0**	**6.47**	**1.17**	**1.99**	**1.21**
**KETONES**						
2-Pentadecanone, 6,10,14-trimethyl	C_18_H_36_O		2.39			
9-(Tetrahydropyran-2-yloxy)-4,6-dioxatricyclo[5.3.1.0(3,8)]undecan-5-one	C_14_H_20_O_5_			1.29		
4,7,7-Trimethyl-5-(tetrahydropyran-2-yloxy)-bicyclo[2.2.1]heptan-2-one	C_15_H_24_O_3_				1.32	
1-Cyclohexene, 1,3,3-trimethyl-2-(1-methylbut-1-en-3-on-1-yl)-	C_14_H_22_O					1.13
**TOTAL KETONES**		**0**	**2.394**	**1.29**	**1.32**	**1.13**
**ALCOHOLS**						
1-Dodecanol, 3,7,11-trimethyl-	C_15_H_32_O		8.69	4.11		17.28
2-Propylcyclohexanol	C_9_H_18_O			2.99		
Phytol	C_20_H_40_O	21.62	40.85	40.03	48	40.14
Behenic alcohol	C_22_H_46_O			1.13		
2-Norpinanol, 3,6,6-trimethyl-	C_10_H_18_O			5.20		
3,7,11,15-Tetramethyl-2-hexadecen-1-ol	C_20_H_40_O				4.29	
**TOTAL ALCOHOLS**		**21.62**	**49.54**	**53.48**	**52.3**	**57.43**
**TOTAL Areas (%)**		**94.28**	**97.81**	**97.14**	**94.62**	**94.3**
**Others Areas (%) = 100-Total Areas (%)**		**5.71**	**2.18**	**2.85**	**5.37**	**5.69**

**Table 6:** Product yield percentages of outstanding hydrocarbons (alkanes and alkenes) and alcohols compounds from catalytic deoxygenation of the algal HO over the parent and Cerium-modified zeolites at 300 °C for 6 h under initial N_2_ pressure of 7 bar, 1000 rpm, and 23.6 g of algal HO/3.54 g of the catalyst in the batch reactor.

Hydrocarbon Compound	Molecular Formula	Hydrolyzed Oil (HO)	HZSM-5	5%Ce/ HZSM-5	10%Ce/ HZSM-5	15%Ce/ HZSM-5
Hexacosane	C_26_H_54_	11.53				
Tetradecane	C_14_H_30_		4.78	4.2	5.21	5.36
Octane	C_8_H_18_			1.05		
Bicyclo[3.1.1]heptane, 2,6,6-trimethyl-, (1.alpha.,2.beta.,5.alpha.)	C_10_H_18_					4.38
5-Ethyl-1-nonene	C_11_H_22_		17.04	17.75	20.65	4.83
1-Undecene, 8-methyl-	C_12_H_24_				4.13	5.1
2-Hexadecene, 2,6,10,14-tetramethyl-	C_20_H_40_					2.49
**The total yield of the hydrocarbon compounds**		**11.53**	**21.83**	**23.02**	**30**	**22.17**
**Alcohol** **Compound**	**Molecular** **formula**	**Algal** **HO**	**HZSM-5**	**5%Ce** **/HZSM-5**	**10%Ce** **/HZSM-5**	**15%Ce** **/HZSM-5**
1-Dodecanol, 3,7,11-trimethyl-	C_15_H_32_O		8.69	4.11		17.28
2-Propylcyclohexanol	C_9_H_18_O			2.99		
Phytol	C_20_H_40_O	21.62	40.85	40.03	48	40.14
Behenic alcohol	C_22_H_46_O			1.13		
2-Norpinanol, 3,6,6-trimethyl-	C_10_H_18_O			5.2		
3,7,11,15-Tetramethyl-2-hexadecen-1-ol	C_20_H_40_O				4.29	
**The total yield of the alcohol compounds**		**21.62**	**49.54**	**53.48**	**52.303**	**57.43**

**Table 7:** Hydrocarbons productions via catalytic deoxygenation with various catalyst types in references and catalytic deoxygenation in this study.

**Reactant**	**Catalyst**	**Reactant/Catalyst** **Ratio**	**Reactant/Solvent**	**Reactor Type**	**Pressure (bar), Gas**	**Temperature (°C)**	**Time (h)**	**Conversion (%)**	**Observations**	Ref.
palm kernel oil	HBeta zeolite	10/1.5	-	B.R	10 bar H_2_	350	5	-	The total yield of hydrocarbons = 82 ± 3%	[8]
Hydrolyzed palm kernel oil	HBeta zeolite	10/1.5	-	B.R	10 bar H_2_	350	5	-	The total yield of hydrocarbons = 24 ± 9%	[8]
Olein oil	HBeta zeolite	10/1.5	-	B.R	10 bar H_2_	350	5	-	The total yield of hydrocarbons = 43 ± 3%	[8]
Hydrolyzed olein oil	HBeta zeolite	10/1.5	-	B.R	10 bar H_2_	350	5	-	The total yield of hydrocarbons = 98 ± 4%	[8]
Hydrolyzed Macauba oil	HBeta zeolite	10/1	-	B.R	10 bar H_2_	350	5	-	The total yield of hydrocarbons = 30%	[8]
Hydrolyzed castor oil	5% Pd/C	1/0.1	1 g Hydrolyzed castor oil/30 mL *n*-hexane	B.R	25 bar H_2_	310	7	-	The total yield of hydrocarbons = 57%	[9]
Hydrolyzed castor oil	5% Pd/C	1/0.1	1 g Hydrolyzed castor oil/30 mL *n*-dodecane	B.R	25 bar H_2_	310	7	-	The total yield of hydrocarbons = 39.6%	[9]
Hydrolyzed castor oil	5% Pd/C	1/0.1	1 g Hydrolyzed castor oil/30 mL *n*-hexane	B.R	25 bar H_2_	300	7	-	The total yield of hydrocarbons = 40%	[9]
Hydrolyzed castor oil	5% Pd/C	1/0.1	1 g Hydrolyzed castor oil/30 mL *n*-hexane	B.R	25 bar H_2_	340	7	-	The total yield of hydrocarbons ~96%	[9]
Stearic acid	10%Ni/HZSM-5 (Si/Al = 40)	1/0.2	1 g stearic acid/ 100 mL dodecane	B.R	40 bar H_2_	260	8		Total selectivity of hydrocarbons ~56%	[10]
Microalgae oil	10%Ni/HBeta (Si/Al =180)	1/0.2	1 g Microalgae oil/100 mL dodecane	B.R	40 bar H_2_	260	6		The total yield of hydrocarbons = 70%	[10]
Crude oil of microalgae	10%Ni/ ZrO_2_	1/0.5	-	B.R	40 bar H_2_	270	6	-	The total yield of hydrocarbons = 72%	[11]
Crude oil of microalgae	10%Ni/ ZrO_2_	1/0.5	-	B.R	40 bar H_2_	270	4	-	The total yield of hydrocarbons = 61%	[11]
Palmitic acid	Ni/LY char	1/1	1 g Palmtic acid/ 10 g hexane	B.R	30 bar H_2_	300	5	31.41	The total yield of hydrocarbons = 12.75%	[12]
Palmitic acid	Ni/LY char	1/1	1 g Palmtic acid/ 10 g acetone	B.R	30 bar H_2_	300	5	67	The total yield of hydrocarbons = 12.49%	[12]
Methyl oleate	5%Pd/C	0.83 mol/L/ 1 g of catalyst	-	Semi-batch	15 bar H_2_	300	6	96	Total selectivity of hydrocarbons = 29%	[13]
Methyl oleate	5%Pd/C	0.83 mol/L/ 1 g of catalyst	-	Semi-batch	15 bar Ar	300	6	44	Total selectivity of hydrocarbons = 17%	[13]
Soybean oil	20%Ni/ Al_2_O_3_	50/0.55	-	B.R	7 bar N_2_	350	4	74	The total yield of hydrocarbons = 79.5%	[86]
Stearic acid	Pd/ Al_2_O_3_	1	-	B.R	7 bar N_2_	350	6	43	Total selectivity of hydrocarbons = 35%	[87]
Cellulose and glycerol	HZSM-5 (Si/Al = 36)	cellulose:glycerol: catalyst = 1:0.05:0.004	100 g of *n*-heptane	B.R	-	350	0.5	-	The total yield of hydrocarbons = 21%	[88]
Cellulose and glycerol	5%Fe/ HZSM-5 (Si/Al = 36)	cellulose:glycerol: catalyst = 1:0.05:0.004	100 g of *n*-heptane	B.R	-	350	0.5	-	The total yield of hydrocarbons = 38%	[88]
Lauric acid	5%Pd/C	1/0.1	1 g of acid/ 100 mL of hexadecane	S.B.R	20 bar Ar	300	6	-	The total yield of hydrocarbons = 38	[89]
Lauric acid	5%Pd/C	1/0.1	1 g of acid/ 100 mL of hexadecane	S.B.R	20 bar Ar	300	3	-	The total yield of hydrocarbons = 28	[89]
Algal HO	HZSM-5 (Si/Al = 30)	1 g of algal HO/0.15 g of the catalyst	-	B.R	7 bar N_2_	300	6	94.58	The total yield of hydrocarbons = 21.83%	This study
Algal HO	5%Ce/ HZSM-5 (Si/Al = 30)	1 g of algal HO/0.15 g of the catalyst	-	B.R	7 bar N_2_	300	6	97.19	The total yield of hydrocarbons = 23.02%	This study
Algal HO	10%Ce/ HZSM-5 (Si/Al = 30)	1 g of algal HO/0.15 g of the catalyst	-	B.R	7 bar N_2_	300	6	98.20	The total yield of hydrocarbons = 30%	This study
Algal HO	15%Ce/ HZSM-5 (Si/Al = 30)	1 g of algal HO/0.15 g of the catalyst	-	B.R	7 bar N_2_	300	6	96.21	The total yield of hydrocarbons = 22.17%	This study

**Table 8:** Alcohol productions via catalytic deoxygenation with various catalyst types in references and catalytic deoxygenation in this study.

Reactant	Catalyst	Operating Conditions	Observation	Ref.
Palmitic acid	Limonite catalyst	The catalytic hydrotreating of the palmitic acid was conducted in a batch reactor with the weight ratio of palmitic acid:catalyst: solvent (hexane) of 1:1:10, respectively at 300 °C, 30 bar of H_2_.	The total yield percentage of alcohol = 51.84 and 38.35 at 5 h and 3 h respectively	[12]
Cellulose, and glycerol	HZSM-5 (Si/Al = 36) and 5%Fe/ HZSM-5 (Si/Al = 36)	Cellulose, glycerol, and catalyst were added to the batch reactor with weight ratios of 1:0.05:0.004 respectively, then 100 g of *n*-heptane was added, and the reaction temperature is 350 °C for 0.5 h	The total yield of alcohol compounds~ 26% using HZSM-5 and about ~20% using 5%Fe/HZSM-5	[92]
Lauric acid	5%Pd/C	1 g of Lauric acid with 100 mL of hexadecane and 0.1 g of catalyst was added to the semi-batch reactor. The deoxygenation reaction temperature was performed at a temperature of 300 °C, and 6 h.	The total yield percentage of alcohol = 9% and 0% using 20 bar of H_2_ and 20 bar of Ar, respectively.	[93]
Stearic acid	4%Ru/ TiO_2_	1 g of stearic acid with 100 mL of dodecane and 0.1 g of catalyst was added to the semi-batch reactor. The deoxygenation reaction temperature was performed at a temperature of 220 °C, and 20 bar of H_2_.	The total yield percentage of alcohol= 20% and 0% at 1 h and 6 h, respectively.	[93]
Stearic acid	4%Re/ TiO_2_	1 g of stearic acid with 100 mL of dodecane and 0.1 g of catalyst was added to the semi-batch reactor. The deoxygenation reaction temperature was performed at a temperature of 220 °C, 6 h, and 20 bar of H_2_.	The total yield percentage (%) of alcohol = 81%	[93]
Soybean oil	35%ɤAl_2_O_3_/CaO	The experiment was conducted under atmospheric pressure in a fixed bed reactor; 6 g of catalyst was placed in the middle of the reactor, then 24 g of soybean oil was injected with WHSV = 3.72 h^−1^ at 480 °C.	The total yield of alcohol compounds = 12.3%	[94]
Sugarcane bagasse	HZSM-5 (Si/Al = 23) and 1%Ce/ HZSM-5 (Si/Al = 23)	The catalytic pyrolysis experiment was conducted under atmospheric pressure in a fixed bed reactor. 1 g of catalyst and 2 g of Sugarcane bagasse were placed in the reactor at 500 °C.	The total yield percentage (%) of alcohol~14% and ~5% using HZSM-5(Si/Al = 23) and 1%Ce/HZSM-5(Si/Al = 23), respectively.	[21]
Algal HO	HZSM-5 (Si/Al = 30), 5%Ce/HZSM-5 (Si/Al = 30), 10%Ce/HZSM-5 (Si/Al = 30), and 15%Ce/HZSM-5 (Si/Al = 30)	The catalytic deoxygenation of the algal HO in the batch reactor at 300 °C, 6 h, 7 bar of initial inert N_2_ gas, 1 g of algal HO/0.15 g of the catalyst.	The total yield percentage (%) of alcohol = 49.54, 53.48, 52.30, and 57.43 using HZSM-5, 5%Ce/HZSM-5, 10%Ce/HZSM-5, and 15%Ce/HZSM-5, respectively.	Current study

**Table 9:** The degree of deoxygenation, elemental composition, higher heating value, H/C, and O/C atomic ratios for the algal HO and the liquid products of the catalytic deoxygenation over the parent HZSM-5 and Cerium modified zeolites catalysts.

NO.	Liquid Type	Element (%)			HHV (MJ/Kg)	H/C (Mole Ratio)	O/C (Mole Ratio)	DOD%
		C	H	O				
1	Algal hydrolyzed oil (HO)	78.91	12.43	8.65	32.37	1.89	0.08	n.a
2	Liquid product for HZSM-5	82.9	12.02	5.06	33.23	1.74	0.04	44.235
3	Liquid product for 5%Ce/HZSM-5	80.66	13.34	5.99	33.48	1.98	0.05	32.22
4	Liquid product for 10%Ce/HZSM-5	82	13.63	4.36	34.05	1.99	0.03	51.44
5	Liquid product for 15%Ce/HZSM-5	81.23	13.61	5.14	33.82	2.01	0.04	42.26
6	Crude oil [93]	83–86	11–14	˂ 1	44	1.5–2	~ 0	n.a

n.a: not applicable.

## Data Availability

Not applicable.

## Supplementary Materials

The following supporting information can be downloaded at: https://www.mdpi.com/article/10.3390/molecules27217251/s1, Figure S1: Scheme of the soxhlet extractor used to extract the crude oil from Chlorella Vulgaris microalgae powder.

## Sample Availability

Samples of the compounds are available from the authors.
