# Supplementary material for: Ce-Loaded HZSM-5 Composite for Catalytic Deoxygenation of Algal Hydrolyzed Oil into Hydrocarbons and Oxygenated Compounds
Source: Molecules. 2022 Oct 25;27(21):7251. doi: 10.3390/molecules27217251 (PMC9658450; doi:10.3390/molecules27217251)
Supplement: Supplementary file 1 [file molecules-27-07251-s001.zip › molecules-1895439-supplementary.pdf]

Ce-Loaded HZSM-5 Composite for Catalytic Deoxygenation of Algal Hydrolyzed Oil into Hydrocarbons and Oxygenated Compounds

Mustafa Jawad Nuhma [1,2], Hajar Alias [1,*], Muhammad Tahir [3,*] and Ali A. Jazie [2]

[1] Department of Chemical Engineering, School of Chemical and Energy Engineering, Universiti Teknologi Malaysia, Johor Bahru 81310, Malaysia.

[2] Chemical Engineering Department, College of Engineering, University of Al-Qadisiyah, Al-Diwaniyah City, P.O. Box 88, Iraq.

[3] Chemical and Petroleum Engineering Department, United Arab Emirates University (UAEU), Al Ain P.O. Box 15551, United Arab Emirates.

* Correspondence: r-hajar@utm.my (H.A.); muhammad.tahir@uaeu.ac.ae (M.T.); Tel.: +60-19-385 5571 (H.A.); +971-509961678 (M.T.)

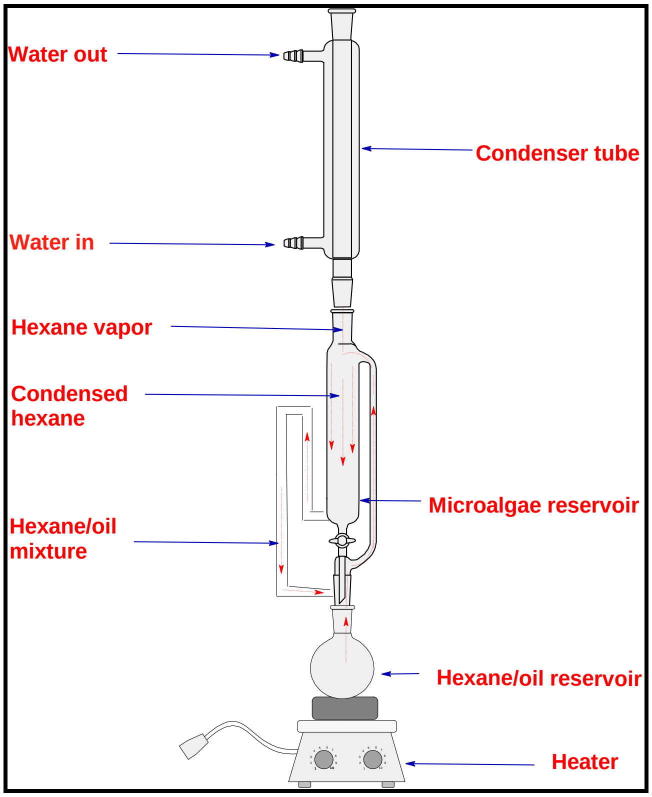

Figure S1. Scheme of the soxhlet extractor used to extract the crude oil from Chlorella Vulgaris microalgae powder.
